# Systematic assessment of the influence of quality of studies on mistletoe in cancer care on the results of a meta-analysis on overall survival

**DOI:** 10.1007/s00432-024-05742-1

**Published:** 2024-04-29

**Authors:** Jorina Hofinger, Lukas Kaesmann, Jens Buentzel, Martin Scharpenberg, Jutta Huebner

**Affiliations:** 1 Klinik für Innere Medizin II, Hämatologie und Onkologie, Universitätsklinikum Jena, Jena, Germany; 2 Klinik und Poliklinik für Strahlentherapie und Radioonkologie, Ludwig-Maximilians-Universität München, Munich, Germany; 3 Klinik für Hals‑Nasen‑Ohren‑Heilkunde, Südharzklinikum, Nordhausen, Germany; 4 Kompetenzzentrum Für Klinische Studien, Universität Bremen, Bremen, Germany

**Keywords:** Cancer, Meta-analysis, Mistletoe therapy, Overall survival, Risk of bias, Methodological quality

## Abstract

**Purpose:**

Mistletoe treatment in cancer patients is controversial, and a Cochrane review concluded that due to heterogeneity, performing a meta-analysis was not suitable. However, several systematic reviews included meta-analyses in favor of mistletoe. The aim of this work was to assess the influence of the methodological quality of controlled studies on the results of a meta-analysis regarding overall survival.

**Methods:**

Between April and August 2022, Medline, Embase, Cochrane Central Register of Controlled Trials (CENTRAL), PsycINFO, CINAHL and Web of Science were systematically searched. In addition, reference lists of previously published meta-analyses were checked for relevant publications. A random effects meta-analysis with clustering was performed. The risk of bias within the studies was assessed using ROB 2.0 and ROBINS-I.

**Results:**

The search identified 4685 hits, and 28 publications reporting on 28 298 patients were included in the quantitative analysis. Overall, the analysis led to a significant result in favor of mistletoe therapy (overall HR = 0.61 with 95% CI [0.53;0.7]). According to our subgroup analysis of randomized studies, studies of higher quality (lower risk of bias) did not lead to a significant result in favor of mistletoe therapy (HR = 0.78; CI = [0.30; 2.00]).

**Conclusions:**

In the case of mistletoe therapy, the results of the meta-analysis strongly depended on the methodological quality of the included studies. Calculating meta-analyses that include low-quality studies may lead to severe misinterpretation of the data.

**Supplementary Information:**

The online version contains supplementary material available at 10.1007/s00432-024-05742-1.

## Introduction

The mistletoe (Viscum album) is a hemiparasite that grows on a variety of host trees in Europe, Asia and North Africa. Extracts of Viscum album are used either as alternative or complementary treatments in cancer therapy (Huebner et al. 2014). The use of mistletoe extracts in cancer therapy originated in anthroposophical medicine and was attributed to Rudolf Steiner and Ita Wegmann (Horneber et al. 2008). In German-speaking countries, mistletoe therapy is one of the most frequently used methods of complementary and alternative medicine in oncology, although the available evidence is still not sufficient for a clear recommendation for or against this therapy (AWMF 2021). Endpoints of interest in relation to mistletoe therapy, which have been investigated in numerous studies over the past decades, are quality of life, reduction of therapy-associated side effects, prolongation of overall survival and disease-free survival, as well as toxicity. In this systematic assessment, the overall survival under mistletoe therapy will be investigated.

As the number of available studies increased, systematic reviews were conducted. These reviews (Freuding 2019; Staupe 2022) draw a rather sceptical conclusion and expressed criticism of the available evidence on mistletoe, even stating that the available literature does not provide any indication to prescribe mistletoe to patients with cancer with respect to overall survival (Freuding 2019). Cochrane review authors (Horneber et al. 2008) and a DIMDI (Deutsches Institut für medizinische Dokumentation und Information)-Investigation (Lange-Lindberg et al. 2006) state that it is impossible to perform meta-analyses concerning mistletoe therapy due to the high heterogeneity of the available data. Horneber et al. (2008) further encourage the conduction of high quality, independent clinical research. Several authors have noted that the calculation of meta-analyses is impossible because most of these studies involve heterogeneous data with a high risk of bias (Huebner et al. 2019; Horneber et al. 2008; Lange-Lindberg et al. 2006).

In the following years, very few studies on overall survival in cancer patients receiving mistletoe therapy were conducted. Nonetheless, meta-analyses have since been calculated and published. In these meta-analyses, some authors propose a survival benefit (Ostermann et al. 2009, 2020; Loef and Walach 2022; Ostermann and Büssing 2012), while other meta-analyses (Ziegler and Grossarth-Maticek 2010) showed differing results in quantitative analysis of randomized (non-significant result) and non-randomized studies (significant result). In evidence-based medicine, a meta-analysis based on randomized controlled trials allows high levels of recommendation to be made. Meta-analyses consequently have a high impact on guidelines such as the German S3 guidelines on complementary medicine in the treatment of oncological patients (AWMF 2021) and set standards for best practice in everyday treatment.

In case of mistletoe therapy, heterogeneity of data as well as a low methodical quality (Lange-Lindberg et al. 2006) are to date the main reasons for questioning the calculation of a meta-analysis. A rigorous assessment of the risk of bias of the included studies with validated tools seems essential, especially as mistletoe research is still dominated by a few large studies by individual research groups (e.g., Grossarth-Maticek et al.).

Therefore, it is necessary to revisit the question of whether it is possible to perform a valid meta-analysis on overall survival in cancer patients receiving mistletoe therapy based on the existing data. It seems plausible that the inclusion of large studies with low methodological quality may bias the results of a quantitative analysis. This phenomenon will be investigated by calculating subgroup-analyses and further discussed.

## Objectives

The aim of this review is to assess the extent to which the inclusion or exclusion of studies of varying methodological quality in a meta-analysis has an impact on the resulting statement on the efficacy of mistletoe therapy in cancer patients regarding overall survival.

## Methods

### Inclusion and exclusion criteria

Inclusion and exclusion of studies took place based on the PICO-scheme. Cancer patients of all ages, with all entities and stages of cancer were included. Studies reporting on primary prevention and precancerous conditions were excluded. No restrictions regarding mistletoe extract, dose, type or mode of application were made. Feasibility studies and grey literature were excluded. All types of controlled studies were included. The outcome which was necessary for inclusion was overall survival. All inclusion and exclusion criteria are listed in e-supplementary Table 1.

### Search strategy

The following databases were searched systematically between April and August 2022: Medline, Embase, Cochrane Central Register of Controlled Trials (CENTRAL), PsycINFO, CINAHL and “Science Citation Index Expanded” (Web of Science). A complex search strategy was developed for each database that combined mesh terms/keywords and text words related to cancer and mistletoe. No restriction regarding study type or publication date was made. Articles published in languages other than German or English were not considered. The exact search strategy for each database is listed in e-supplementary Table 2. In addition, reference lists of previously published meta-analyses on survival in cancer patients treated with mistletoe extracts (Ostermann et al. 2009, 2020) and further meta-analyses on mistletoe treatment (Pelzer et al. 2022, 2021; Loef and Walach 2020) were checked for further relevant publications.

### Selection of publications and data management

**Fig. 1:**
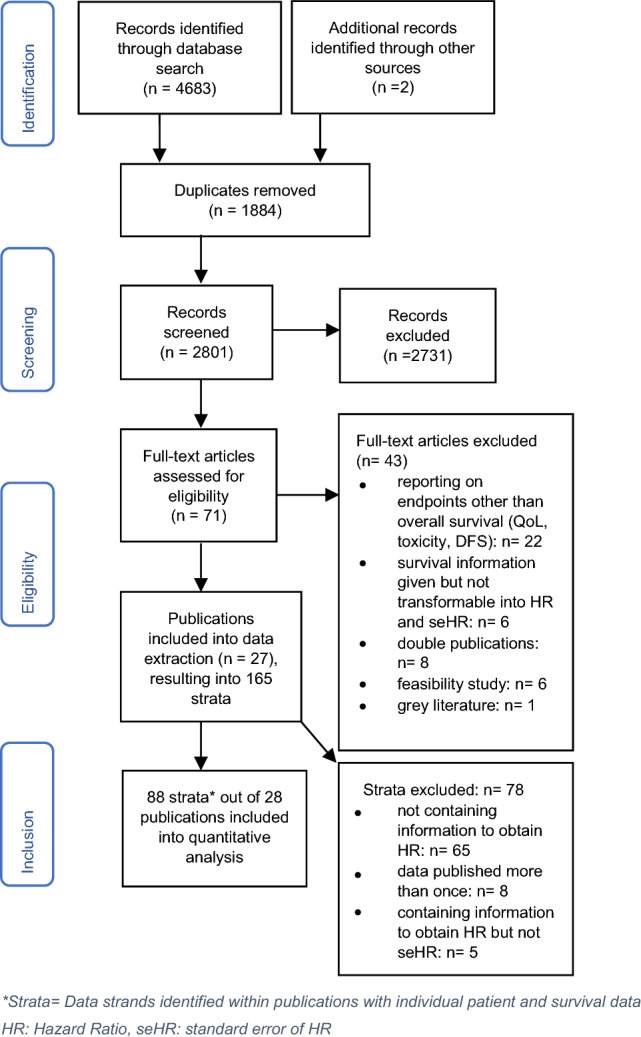
PRISMA (2009). *Strata= Data strands identified within publications with individual patient and survival data, HR: Hazard Ratio, seHR: standard error of HR

After the systematic search and removal of duplicates, all the publications were screened by title and abstract independently by JHo and JHu who assessed each study for relevance according to the inclusion and exclusion criteria. The full-texts of all studies classified as “possibly relevant” were examined and if eligible, the data were extracted by JHo and JHu independently. In cases of disagreement, a consensus was reached by discussion. The data collected in a table included the publication title, date, authors, country the study was conducted in, cancer type and stage, grade, sample size, age, sex, dropouts, funding sources, conventional therapies applied, mistletoe preparation, duration and treatment mode of mistletoe therapy, adverse events after treatment, study design and survival data. Where survival data was not given even though overall survival was an endpoint of the study, we contacted authors via email. For further details on the selection of studies see Fig. 1 following PRISMA (Moher et al. 2009).

### Studies, subgroups, and strata

Several *publications* reported on more than one study. In this context, a *study* is understood as a separate clinical experiment or a retrospective observation for which the population, methodology and results have been described. Within the studies, there were often several *subgroups*, meaning smaller groups of a population (e.g., separated by stage, tumor entity or treatment), for which separate results were reported. Since both full studies and subgroups provided outcome data on overall survival, they were all included in the data table. To maintain clarity, only the terms publication, study and subgroup were used. To summarize the data sets found in publications, we further refer to both full studies and subgroups as “strata”.

### Assessment of the risk of bias of the included studies

First, studies were sorted into randomized and non-randomized studies. Second, non-randomized studies were assessed regarding their risk of bias using the Cochrane risk of bias tool ROBINS-I (Sterne et al. 2016) by JHu and JHo independently. Randomized studies were assessed regarding their risk of bias using the Cochrane tool ROB 2.0 which is specifically designed for risk of bias assessments of randomized studies (Higgins et al. 2022). Both instruments have been published with a cribsheet. These cribsheets were used to reproducibly assess the risk of bias of each study in various categories (ROB 2.0: Risk of bias arising from randomization process, effect of assignment to intervention, effect of adhering to intervention, risk of bias due to missing outcome data, risk of bias in measurement of the outcome, risk of bias in selection of the reported result, overall risk of bias. ROBINS-I: Bias due to confounding, bias in selection of participants into the study, bias in classification of interventions, bias due to deviations from intended interventions, bias due to missing data, bias in measurement of outcomes, bias in selection of the reported result, overall bias) In case of discrepancies, consensus was again reached by discussion.

### Statistical analysis

As the reporting of survival data differed between strata, for inclusion in a meta-analysis, data were extracted, entered into a table and converted into logarithmic hazard ratios (logHRs) and standard errors (seHRs) using the spreadsheet of Tierney et al. (2007). Some strata already reported survival data as HR; in this case, only transformation into the logarithmic form was necessary. If survival data consisted of survival curves, the Tierney method and spreadsheet were used to estimate the hazard ratio (HR). Meta-analysis was performed using the metagen function R 4.2.2 and RStudio 2022.12.0. The metagen function provides the generic inverse variance method for meta-analysis which requires treatment estimates and their standard errors (Borenstein et al. 2010). The presence of multiple strata in a single study or publication was considered by performing a meta-analysis with clusters. JHo and JHu manually determined the clusters independently of each other by assigning numbers to indicate that several subgroups belonged to one study. The results of the meta-analyses are displayed in forest plots. A hazard ratio (HR) < 1 indicates the superiority of the intervention group (mistletoe therapy), while a HR > 1 corresponds to the superiority of the control group. *P* values < 0.05 were considered to indicate statistical significance. In addition, heterogeneity between trials was assessed using the chi^2^ test as well as the Tau(*τ*)^2^ and *I*^2^ coefficients for quantification. The chi^2^ test is calculated using the Q statistics and assumes the null hypothesis that all the studies are homogeneous. If the p value testing this hypothesis is below 0.05, one can conclude that heterogeneity is present (Higgins et al. 2023). *τ*^2^ quantifies the variance of the true effect sizes. *I*^2^ is defined as the percentage of variability in the effect sizes that is not caused by sampling error. *I*^2^ ≤ 25% indicates low heterogeneity, *I*^2^ between 25 and 75% indicates moderate heterogeneity and *I*^2^ ≥ 75% indicates substantial heterogeneity (Harrer et al. 2021).

## Results

The search identified 4685 hits. After removal of duplicates, 2801 records were entered into the title–abstract–screening. Of these, 71 full texts were assessed for eligibility, which led to the inclusion of 28 publications (published between 1966 and 2020) in the quantitative analysis of survival data. The publications of Grossarth-Maticek et al. regularly reported on more than one study. In detail, within 9 publications, 18 eligible studies could be identified. In addition, Günczler et al. (1969) reported on several studies, two of which could be used for quantitative analysis. Altogether, this led to the inclusion of 88 strata extracted from 39 studies published within 28 publications. For details, see Fig. 1.

### Description of included studies

The 88 strata identified within the included studies reported a total of 34,262 patients. It is important to note that some studies published survival data for both the full population and subgroups, which led to multiple inclusions of patients in the meta-analyses. This was considered by clustering the meta-analyses. When counting every patient just once, 28,298 individuals remained. The sample size ranged from 19 (Longhi et al. 2020a, b) to 18 528 (Fritz et al. 2018) patients, with a median of 111 (interquartile range 60.25–204). The median age of the patients was 55.66 years (IR: 51.66–60.2). A total of 83.9% of patients were reported to be female. 14 studies were randomized, and 25 were not. No study was blinded. A total of 29 studies used a prospective design, five studies used a retrospective design, and three used a retrolective design. By “retrolective”, the authors of the respective studies understand retrospective data analysis from patient records that are chosen without the evaluators knowing neither the outcome of the respective case nor the identity of the respective patient (Augustin et al. 2005; Martin 2005). Three studies reported on prospective cases compared with a retrospective control group. 15 studies used mistletoe therapy (s.c.) as an intervention, while 24 studies were not interventional/only observed patients or suggested mistletoe therapy. The studies of Grossarth-Maticek et al. all used a matched pair design, whereas no other studies did. For further details on the study characteristics, please see Table 1.

**Table 1:** Study characteristics

Author, study, year, references	Strata	*n* case/control	Country	Cancer	Funding	Rand	Status	Timeline
Augustin (2005)	1	329/357	DE, CH	Skin	Foundational	No	Observational	Retrolective
Dold (1991)	1	114/113	DE	Lung	Public/Institutional	Yes	Interventional	Prospective
Fellmer (1966)	2	81/800	DE	Gynecological	Foundational	No	Interventional	Prospective
Fritz (2018)	2	164/18364	DE	Breast	Foundational	No	Observational	Retrospective
Grossarth-Maticek CORPUS (2008)	3	198/198	DE	Gynecological	Foundational and public	No	*	Prospective
Grossarth-Maticek CORPUSRAND (2008)	3	56/56	DE	Gynecological	Foundational and public	Yes	*	Prospective
Grossarth-Maticek OVAR (2007a)	3	137/137	DE	Gynecological	Foundational and public	No	*	Prospective
Grossarth-Maticek OVARRAND (2007a)	3	41/41	DE	Gynecological	Foundational and public	Yes	*	Prospective
Grossarth-Maticek CervixMetRand (2007b)	1	19/19	DE	Gynecological	Foundational and public	Yes	*	Prospective
Grossarth-Maticek CERVIX (2007b)	3	168/168	DE	Gynecological	Foundational and public	No	*	Prospective
Grossarth-Maticek Melanoma (2007c)	1	32/32	DE	Skin	Not reported	No	*	Prospective
Grossarth-Maticek MelanomaRand (2007c)	1	22/22	DE	Skin	Not reported	Yes	*	Prospective
Grossarth-Maticek Mamma (2006a)	1	84/84	DE	Breast	Foundational and public	No	*	Prospective
Grossarth-Maticek MammaRand (2006a)	1	38/38	DE	Breast	Foundational and public	Yes	*	Prospective
Grossarth-Maticek MammaRec (2006b)	1	42/42	DE	Breast	Foundational and public	No	*	Prospective
Grossarth-Maticek MammaLym (2006b)	1	55/55	DE	Breast	Foundational and public	No	*	Prospective
Grossarth-Maticek MammaMet (2006b)	1	83/83	DE	Breast	Foundational and public	No	*	Prospective
Grossarth-Maticek MammaLymRand (2006b)	1	17/17	DE	Breast	Foundational and public	Yes	*	Prospective
Grossarth-Maticek (2004)	1	49/49	DE	Multiple	Foundational and public	Yes	*	Prospective
Grossarth-Maticek Full population strict (2001)	1	396/396	DE	Multiple	Foundational and public	No	*	Prospective
Grossarth-Maticek Rand balanced (2001)	1	49/49	DE	Multiple	Foundational and public	Yes	*	Prospective
Grossarth-Maticek Subgroups cancer type (2001)	5	276/276	DE	Multiple	Foundational and public	No	*	Prospective
Günczler Mamma (1969)	2	257/153	AT	Breast	Not reported	No	Interventional	Retrospect. control group
Günczler Colon (1969)	1	47/91	AT	Gastrointestinal	Not reported	No	Interventional	Prospective
Günczler (1968)	4	67/101	AT	Gastrointestinal	Not reported	No	Interventional	Prospective
Hassauer (1979)	4	25/22	DE	Gynecological	Not reported	No	Interventional	Retrospect. control group
Hoffmann (1982)	6	254/241	DE, CH	Breast	Not reported	No	Interventional	Retrospect. control group
Hoffmann(1979)	2	188/122	DE, CH	Multiple	Not reported	No	Interventional	Retrospect. control group
Kleeberg (2004)	3	102/102	**	Skin	Public and corporate	Yes	Interventional	Prospective
Leroi (1977)	2	319/228	CH	Breast	Not reported	No	Observational	Retrospective
Leroi (1975)	1	81/30	DE, CH	Breast	Not reported	No	Interventional	No Information
Longhi (2020a, b)	1	9/10	IT	Osteosarcoma	None	Yes	Interventional	Prospective
Matthes (2010)	5	201/195	DE, CH	Gastrointestinal	Foundational	No	Observational	Retrolective
Salzer (1991)	4	86/97	DE, AT	Lung	Not reported	Yes	Interventional	Prospective
Salzer (1985)	6	37/40	AT	Lung	Corporate	No	Observational	Retrospective
Salzer (1983)	2	62/75	AT	Gastrointestinal	Not reported	Yes	Interventional	Prospective
Schmidt (2007)	1	710/732	DE	Breast	Not reported	No	Observational	Retrolective
Schuppli (1990)	1	84/114	CH	Skin	Not reported	No	Interventional	Prospective
Tröger (2013)	5	110/110	RS	Gastrointestinal	Foundational	Yes	Interventional	Prospective

*Strata*  data strands identified within publications with individual patient and survival data, *Gynecological* *Cancer*  Other than Breast Cancer, *Rand*  randomization

^*^Intervention consisted of suggesting that the patient discuss mistletoe therapy with their doctor

^**^Countries in which the conduction of the study of Kleeberg (2004) took place: DE, FR, CH, AT, BE, UK, YU, IL, CZ, EE, GR, SP, and PL

### Excluded studies

The references of 43 excluded publications are cited in the file e-supplementary Table 3. Furthermore, strata that had to be excluded even though they reported on overall survival as they did not report survival data in eligible ways and the results of these strata can be found in e-supplementary Table 3.

### Risk of bias

Table 2 presents the risk of bias for randomized studies as assessed with the use of the Cochrane risk of bias tool ROB 2.0 (Higgins et al. 2022). Possible outcomes for the different domains were low risk of bias, some risk of bias or high risk of bias.

**Table 2:** Risk of bias in randomized studies

Author	Year	Random sequence generation	Effect of assignment to intervention	Effect of adhering to intervention	Missing outcome data	Measurement of the outcome	Selection of the reported result	Overall risk of bis
Dold	1991	Low	High	High	Low	Some	High	High
Grossarth-Maticek CORPUSRAND	2008	High	Some	High	Low	Some	High	High
Grossarth-Maticek OVARRAND	2007c	Some	Some	High	Low	Some	High	High
Grossarth-Maticek CervixMetRand	2007b	Some	Some	High	Low	Some	High	High
Grossarth-Maticek MelanomRand	2007a	Some	High	High	Low	Some	High	High
Grossarth-Maticek MammaRAND	2006b	Some	High	High	Low	Some	High	high
Grossarth-Maticek MammaLymRand	2006a	Some	High	High	Low	Some	High	High
Grossarth-Maticek	2004	Some	High	High	Some	Some	High	High
Grossarth-Maticek	2001	Some	High	High	Some	Some	High	High
Kleeberg	2004	Low	Some	Low	Low	Low	Some	Some
Longhi	2020	Low	High	High	Low	Some	Some	High
Salzer	1991	Low	High	High	Low	Some	Some	High
Salzer	1983	Low	High	High	High	some	High	High
Tröger	2013	Low	Low	Some	Low	Some	Some	Some

Among the 14 randomized studies, 12 had a high overall risk of bias. Only the studies of Kleeberg et al. (2004) and Tröger et al. (2013) were classified with some risk of overall bias. In the following, subcategories of risk of bias of randomized studies will be specified.

The risk of bias within random sequence generation was judged as low or moderate for all 13 randomized studies. As none of the studies were blinded and both participants and carers were aware of participants’ assigned intervention, risk of bias was present due to the effect of assignment to intervention or due to the effect of adhering to intervention within all studies. It must be noted that for some studies (e.g., those of Grossarth-Maticek et al.), the intervention only consisted of suggesting the option of mistletoe therapy to participants randomized into the mistletoe group, and it was furthermore not specified whether patients received mistletoe therapy. The reasons for missing outcome data were generally well described for the randomized studies.

The only publication with a high risk of bias due to missing outcome data is the study of Salzer et al. (1983), where a high dropout rate (nearly one-third of the participants) occurred. In this study, patients were randomized after telephone calls. Therefore, some patients were randomized despite not fulfilling the inclusion criteria. Moreover, some patients were not assessed as planned during the study and were accordingly excluded. In addition, a high number of patients who did not receive the planned treatment were excluded. In sum, 38 of 145 patients in the control group without adjuvant treatment were excluded—36 of 106 patients with planned adjuvant chemotherapy (active control group) and 43 of 108 patients with adjuvant therapy only were excluded. In addition, no statistical analysis was performed for patients who underwent palliative surgery or had stage I gastric carcinoma even though these patients were included in the study at the beginning of the study.

As the outcome “overall survival” can be objectively measured by investigating the participants’ date of death, this was usually performed appropriately. Grossarth-Maticek et al. state that the ultimate follow-up of survival time was performed by investigations at residents’ registration offices and local boards of health. This method can be questioned because the group reports on a cohort of more than 30,000 cancer patients in several publications making such an investigation very difficult and expensive. Due to the unblinded study design of all included randomized studies, there was an innately moderate risk of bias within the measurement of the outcome. All 13 studies were determined to contain a high risk of bias due to selective reporting of the results. While analyses were often planned in detail, only a portion of the planned parameters or subgroups were reported in the publications.

Table 3 presents the risk of bias for nonrandomized studies assessed with the use of the Cochrane Risk of Bias tool ROBINS-I (Sterne et al. 2016). Possible outcomes for the different domains were low risk of bias, moderate risk of bias, serious risk of bias, critical risk of bias and no information on risk of bias.

**Table 3:** Risk of bias in nonrandomized studies

Author	Year	Risk of confounding	Selection of participants	Classification of intervention	Deviations from intended intervention	Missing outcome data	Measurement of the outcome	Selection of reported results	Overall risk of bias
Augustin	2005	Moderate	Serious	Moderate	NI	Moderate	Moderate	moderate	serious
Fellmer	1966	Moderate	Moderate	Moderate	moderate	Serious	Moderate	serious	serious
Fritz	2018	Moderate	Moderate	Moderate	NI	Low	Moderate	Serious	Serious
Grossarth-Maticek CORPUS	2008	Serious	Moderate	Serious	serious	Moderate	Moderate	Serious	Serious
Grossarth-Maticek OVAR	2007c	Serious	Moderate	Serious	serious	Moderate	Moderate	Serious	Serious
Grossarth-Maticek CERVIX	2007b	Serious	Moderate	Serious	serious	Moderate	Moderate	Serious	Serious
Grossarth-Maticek Melanoma	2007a	Moderate	Low	Serious	serious	Serious	Moderate	Serious	Serious
Grossarth-Maticek Mamma	2006b	Moderate	Low	Serious	critical	Serious	Moderate	Serious	Critical
Grossarth-Maticek MammaRec	2006a	Moderate	Serious	Serious	serious	Moderate	Moderate	Serious	Serious
Grossarth-Maticek MammaLym	2006a	Moderate	Moderate	Serious	serious	Moderate	Moderate	Serious	Serious
Grossarth-Maticek MammaMet	2006a	Moderate	Moderate	Serious	serious	Moderate	Moderate	Serious	Serious
Grossarth-Maticek Full population strict	2001	Serious	Moderate	Serious	NI	Serious	Moderate	Serious	Serious
Grossarth-Maticek Subgroups cancer type	2001	Serious	Moderate	Serious	NI	Serious	Moderate	Serious	Serious
Günczler Mamma	1969	Serious	Serious	Low	serious	Moderate	Moderate	Moderate	Serious
Günczler Colon	1969	NI	NI	NI	NI	NI	NI	NI	NI
Günczler	1968	Serious	Low	Moderate	serious	Serious	Moderate	Low	Serious
Hassauer	1979	Serious	Moderate	Moderate	serious	Serious	Moderate	Serious	Serious
Hoffmann	1982	Serious	Serious	Moderate	serious	Low	Moderate	Low	Serious
Hoffmann	1979	Serious	Serious	Moderate	serious	NI	Moderate	Moderate	Serious
Leroi	1977	Serious	Serious	Moderate	NI	Serious	Moderate	Serious	Critical
Leroi*	1975	NI	NI	NI	NI	NI	NI	NI	NI
Matthes	2010	Low	Moderate	Low	NI	Moderate	Moderate	Serious	Serious
Salzer	1985	Moderate	Low	Moderate	NI	NI	Moderate	Moderate	Moderate
Schmidt	2007	Low	Moderate	Moderate	NI	Moderate	Moderate	Moderate	Moderate
Schuppli	1990	Serious	Moderate	Low	NI	NI	Moderate	Moderate	Serious

*NI* No information on risk of bias

Of the 25 nonrandomized studies, 21 were considered to contain a serious or critical risk of overall bias. The nonrandomized studies of Salzer et al. (1985) and Schmidt et al. (2007) were the only ones rated as having a moderate risk of overall bias. Leroi et al. (1975) and Günczler et al. (1969)—subgroup colon cancer—could not be rated with the ROBINS-I tool (Sterne et al. 2016), as the publications do not provide proper information on the study design. Observational studies (Augustin et al. 2005; Fritz et al. 2018; Leroi 1977; Matthes et al. 2010; Salzer 1985; Schmidt and Edgar 2007) could not be assessed with regard to the intervention parameter (risk of bias due to deviations from intended interventions) and therefore received a rating of “4—no information”. Specific findings regarding the individual studies are presented in more detail below.

First, in most studies, there was no prespecified analysis plan, and the results were not reported for all the subgroups included.

While the study of Augustin et al. (2005) controlled for many confounders, it also violated the protocol regarding the planned analyses: The authors announced the calculation of an intention-to-treat analysis regarding the second outcome of the study, safety. This analysis cannot be found within the publication. Instead, a per-protocol analysis is presented. Furthermore, the arm (intervention or control) to which the 52 individuals that were excluded belonged was not specified.

Fellmer et al. (1966) assigned participants to groups according to their last name after they had been initially selected by a senior physician. In the adjusted control group, the authors excluded 91 patients who died during the first months of follow-up. A resulting bias cannot be ruled out.

Fritz et al. (2018) selected 423 controls out of 18.364 available patient records and compared these records with 141 patients treated with mistletoe therapy, which were selected from a cohort of 164 individuals. According to the authors, this process followed a similar case method. Furthermore, the authors calculated many statistical analyses for overall survival, but as they published confidence intervals and *p* values without an actual effect parameter, most of these results could not be used for quantitative analysis.

In the study of Matthes et al. (2010), baseline treatment and tumor stages differed significantly,; therefore, they used adjusted calculations. Unfortunately, this was not the case in most nonrandomized studies, depicting another source of risk of bias.

Among the ten nonrandomized studies of Grossarth-Maticek et al. included, the intervention consisted of suggesting the option of mistletoe therapy to case group participants and it was not specified whether patients received mistletoe therapy. Furthermore, the authors used a matched-pair approach and often reported on balanced and strictly matched pairs separately. Many results are reported as the mean survival time in years without standard deviation and could therefore not be included in the quantitative meta-analysis. Furthermore, it was specified by the authors that the studies commenced in the 1970s and therefore did not have a written study protocol or an initial sample size calculation.

A similar problem occurred with other studies published in the second half of the twentieth century. Günczler (1969) (subgroup mammary carcinoma) and Hassauer (1979) used historical and external control groups, respectively, leading to considerable confounding bias. In addition, Leroi et al. (1977) and Hoffmann et al. (1979, 1982) formed or extended their control group with dropouts from the intervention group who were inadequately treated with mistletoe.

### Meta-analysis of overall survival

**Fig. 2:**
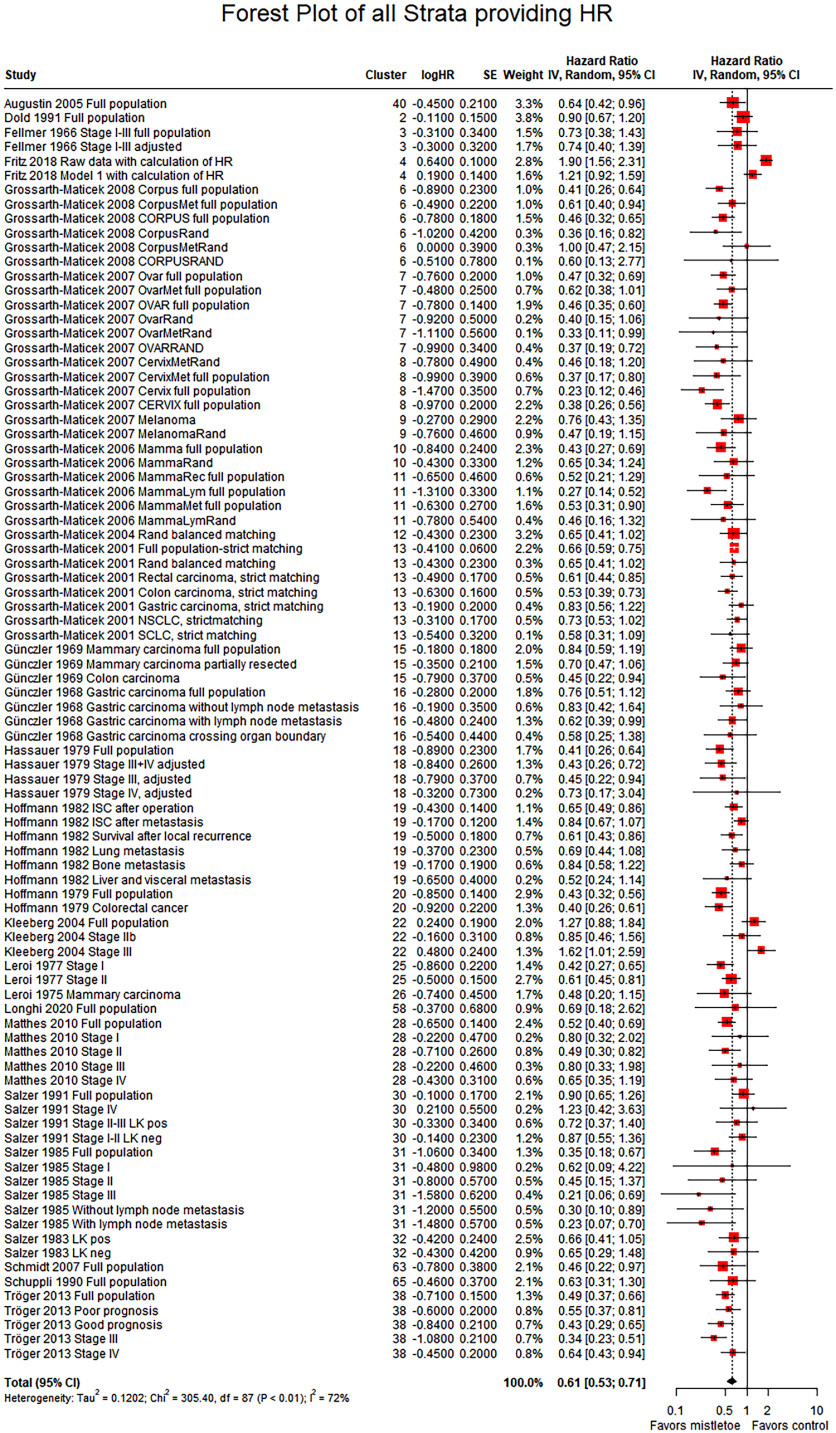
Meta-analysis of all strata providing HR

**Fig. 3:**
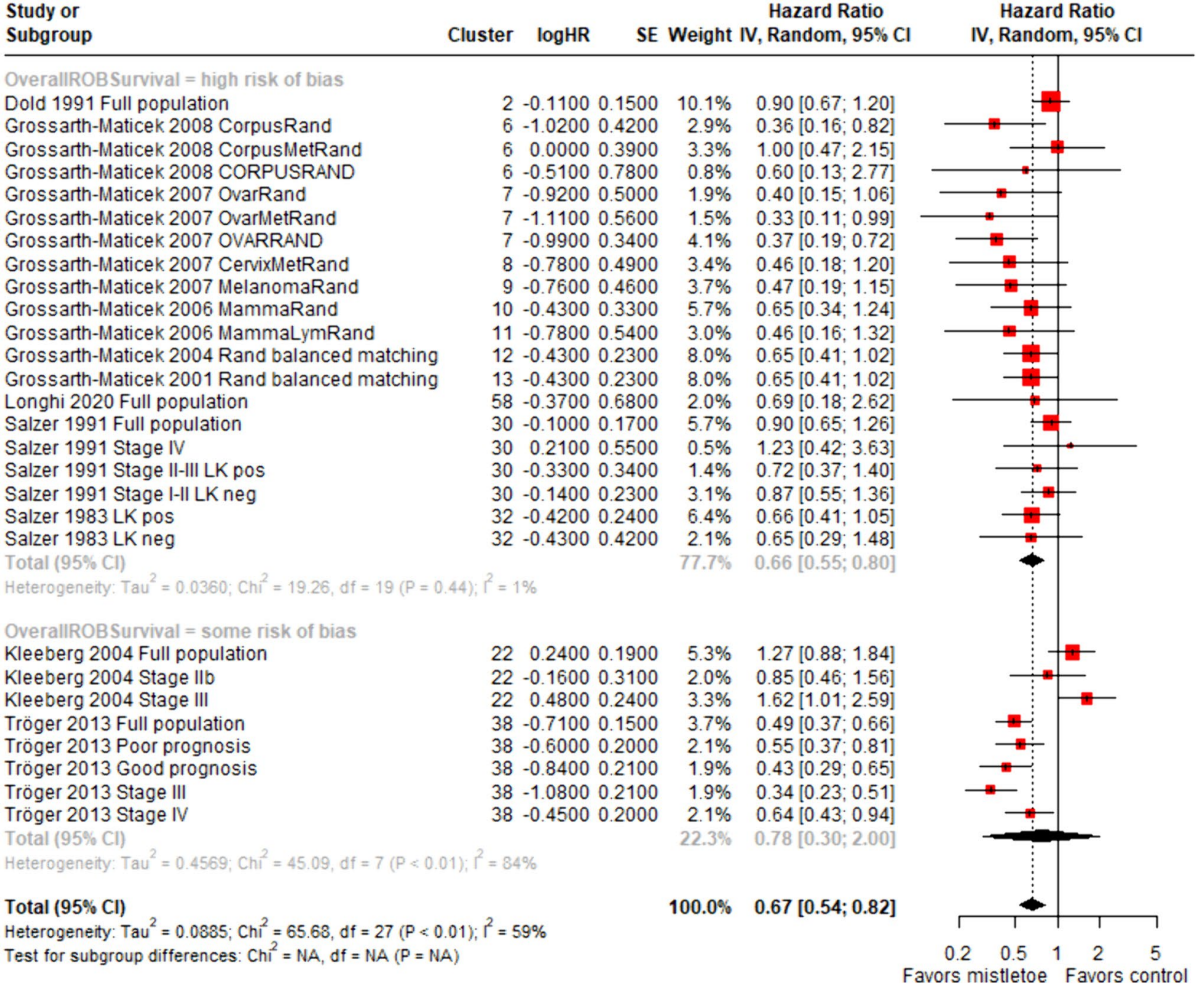
Meta-analysis of all randomized strata stratified by risk of bias

In total, we performed 23 meta-analyses and subgroup analyses, which can be found in detail in the e-supplement. Meta-analysis of all strata providing data eligible for quantitative analysis led to a significant result in favor of mistletoe therapy (overall hazard ratio (HR) = 0.61 with 95% CI [0.53; 0.71]), similar to previously published meta-analyses (Ostermann et al. 2020). Heterogeneity of the study results was substantial and significant (*I*^2^ = 72%, *p* of Chi^2^ test < 0.01), with a between-study variance (*τ*^2^) of 0.1202. Sources of heterogeneity appear to be the differing study designs, differing population sizes and types of intervention as homogeneity occurs as soon as these variables are considered individually in subgroup analyses. Subgroup analyses of contextual variables (tumor type, stage, etc.) mostly aligned with the overall meta-analysis in showing a significant result in favor of mistletoe therapy. Subgroup analysis of strata sorted by risk of bias revealed the following results: In the subgroup analysis of randomized strata, studies of higher quality (lower risk of bias) had nonsignificant results (HR = 0.78; CI = [0.30; 2.00]) but high heterogeneity. The randomized trials that had been classified into a lower quality group were significantly in favor of mistletoe therapy (HR = 0.66; CI = [0.55; 0.80]). Subgroup analysis of nonrandomized studies again revealed positive effects in all subcategories. For further details, please see Figs. 2 and 3.

## Discussion

The aim of this work was to evaluate the influence of contextual and methodological variables on the outcome of a meta-analysis of overall survival under mistletoe therapy. While Lange-Lindberg et al. (2006), commissioned by the DIMDI (Deutsches Institut für Medizinische Dokumentation und Information (German Institute for Medical Documentation and Information), and the Cochrane author group around Horneber et al. (2008) came to the conclusion that the available data are not sufficient to calculate quantitative analyses, other authors (Ostermann et al. 2009, 2020; Loef and Walach 2020; Ziegler and Grossarth-Maticek 2010) have provided meta-analyses in recent years. While the former authors draw a rather skeptical conclusion on the evidence on mistletoe in cancer care, the authors of the meta-analyses came to a more favorable result. Previously, Ziegler et al. (2010) showed that studies of higher methodological quality (randomized) have no significant effect on survival under mistletoe therapy, while studies of lower methodological quality (nonrandomized) have produced statistically significant results. In alignment with our results, we must therefore conclude that the positive results reported by some authors in their meta-analyses are largely based on studies of poor methodological quality.

In our study, we conducted 23 meta-analyses on all strata as well as different subgroups. Most of these subgroups were contextual in nature (e.g., tumor type and early vs. advanced tumor stages), but we also conducted several subgroup analyses concerning methodical questions. The numeric result of the meta-analysis including all eligible strata showed an HR of 0.61 (95% CI: [0.53; 0.71]). The contextual subgroup analyses mostly yielded significant results in favor of mistletoe therapy, in line with the overall analysis of all strata. However, the methodologically oriented subanalyses showed a different result. We categorized the strata according to their risk of bias using the two Cochrane tools “RoB 2.0” and “ROBINS-I” for this purpose. The meta-analysis of the randomized studies according to their risk of bias showed a nonsignificant result for studies with a lower risk of bias (HR = 0.78; CI = [0.30; 2.00]), while studies with a high risk of bias were significantly in favor of mistletoe therapy (HR = 0.66; CI = [0.55; 0.80]). We were not able to reproduce this effect for the nonrandomized studies, as these studies almost all have a higher risk of bias. We assessed all nonrandomized studies with the ROBINS-I tool as suggested by the NICE guidelines ﻿(NICE 2024) This tool has been designed for interventional studies, but the NICE guidelines suggest the use of the ROBINS tool for nonrandomized studies to make the results of risk-of-bias assessments more comparable. In contrast, Ostermann et al. (2020) used the Newcastle Ottawa Scale (Wells et al. 2013) to assess the risk of bias in nonrandomized studies, which led to a judgement of a less severe bias within the studies. It is therefore important to specifically validate instruments or guidelines for assessing the risk of bias for complementary and alternative medicine to address the great variety of study types.

In our analysis, studies of higher methodological quality do not provide significant results in favor of mistletoe therapy with respect to overall survival. Consequently, the inclusion of studies of lower methodological quality in a meta-analysis distorts the result in the direction of significance while simultaneously suggesting a high level of evidence. This result is highly clinically relevant, as guidelines such as the S3 guidelines on complementary medicine in the treatment of oncological patients (AWMF 2021) base their recommendations on Level I evidence, preferably on meta-analyses, and day-to-day treatment decisions are made based on the results of meta-analyses. Research dominated by few or only one group of researchers with a vast impact on the meta-analysis, especially providing studies of lower quality, may entail false treatment recommendations.

In the special setting of research on mistletoe, additional concerns arose: a large number of participants took part in studies from one group of authors (Grossarth-Maticek et al.) In these studies, randomization was not between mistletoe and no mistletoe but between recommendation of mistletoe and no recommendation without knowing the actual treatment status of the patient. These studies dominated previously published meta-analyses. Another concern arose from data sets being published several times without authors clearly stating so. This may lead to a heightened weight of data sets and to results being included more than once. Furthermore, the inclusion of older publications that were not conducted under current methodological standards (e.g., creating control groups out of dropouts) yields an especially high risk of bias, which cannot be adequately taken into account using the RoB/ROBINS tool, as these tools do not consider such methods. It should therefore be kept in mind that some studies could be considerably more biased than could be depicted with the tools used. In addition, the inclusion of studies in meta-analyses require the reporting of results in adequate numerical form. Many strata we had to exclude did not provide these data. This also accounts for recent publications that we had to miss accordingly for our analysis (Seifert et al. 2022). In the special case of this recent publication, an analysis of overall survival took place, but the authors only reported that the result was nonsignificant, withholding the results from being included in the meta-analysis by not publishing them in numerical form. We therefore judged the risk of bias due to selective reporting of results of this publication as high. As we did assess the further risk of bias domains with low or moderate risk of bias, the study by design was of high quality. This therefore is a study of good methodical quality that does not favor mistletoe therapy and is not available for inclusion in the meta-analysis. In addition, it is sometimes not clear how many patients were included in which subgroup [e.g., Matthes (2010), Kleeberg (2004)], making it difficult to include these subgroups in quantitative analysis and to assess risk of bias due to lack of outcome data.

A strict and critical RoB assessment is crucial, especially in the field of complementary and alternative medicine, as the rapidly increasing number of meta-analyses in this field suggests a high level of evidence, and most readers, especially those active in patient care, do not have the time to assess the validity of the results by assessing all the included studies on their own. The importance of this approach has recently been shown in an article about a meta-analysis on homeopathy for ADHD in children (Gaertner et al. 2022). In the case of this article, the paper was withdrawn after more than a year, during which a group of authors around Ernst, Aust and Endruscheit repeatedly noted methodological deficiencies. One of the concerns was a misjudgment of the risk of bias, which led to a distortion of the results (Aust et al. 2023).

### Limitations

Several limitations must be considered with respect to our meta-analyses. First, there was high heterogeneity between the studies included. Second, we had to exclude several articles due to incomplete reporting of statistical data in the publication, and the authors did not provide these data on request. Third, we followed the NICE recommendation and used the Cochrane ROBINS-I tool to assess the risk of bias in noninterventional studies to maximize the comparability of the assessments even though the tool was not developed for non-interventional studies. In addition, we included only publications written in English or German, and we did not consider gray literature.

## Conclusion

In the case of mistletoe, which is a CAM topic with many clinical studies, we have shown that the results of meta-analyses depend on the methodological quality of the included studies. Calculating meta-analyses that include low-quality studies may lead to severe misinterpretation of the data, and the formal level at which evidence is generated may strongly affect the conclusions and recommendations.

In accordance with the Cochrane guidelines (Higgins et al. 2023), as long as there are insufficient data with a low risk of bias and low heterogeneity, a meta-analysis should not be performed. When calculating a meta-analysis, nonetheless, inclusion and exclusion criteria must be reasonably set and strictly adhered to. Furthermore, the results must be communicated with high transparency. Subgroup analysis of studies of high methodological quality should be performed to evaluate the influence of methodological variables.

### Supplementary Information

Below is the link to the electronic supplementary material.

Supplementary file1 (DOCX 14 KB)

Supplementary file2 (DOCX 16 KB)

Supplementary file3 (DOCX 62 KB)

Supplementary file4 (DOCX 1204 KB)

## Data Availability

All the data in our systematic review were derived from published studies and thus are available. On reasonable request, they can be provided by the first author.
