# Supplementary material for: Systematic assessment of the influence of quality of studies on mistletoe in cancer care on the results of a meta-analysis on overall survival
Source: J Cancer Res Clin Oncol. 2024 Apr 29;150(4):219. doi: 10.1007/s00432-024-05742-1 (PMC11056339; doi:10.1007/s00432-024-05742-1)
Supplement: Supplementary file 1 — Supplementary file1 (DOCX 14 KB) [file 432_2024_5742_MOESM1_ESM.docx]

Supplementary plots of meta-analysis corresponding to the article:

# Systematic assessment of the influence of quality of studies on mistletoe in cancer care on the results of a meta-analysis on overall survival

Jorina Hofinger, University of Jena, Klinik für Innere Medizin II, Jena, Germany.

[jorina.hofinger@outlook.de](mailto:jorina.hofinger@outlook.de), corresponding author, ORCID-ID 0009-0007-7169-3915

Lukas Kaesmann, Jens Buentzel, Martin Scharpenberg, Jutta Huebner

Table 1: PICO-Scheme of inclusion and exclusion criteria

| PICO | Inclusion criteria | Exclusion criteria |
| --- | --- | --- |
| Patients | Cancer patients (all ages, entities, and stages) | Primary prevention,  precancerous conditions |
| Intervention | Application and observation of mistletoe extract, no restrictions regarding mistletoe extract, dose, mode of application | Feasibility studies |
| Comparison | All possible control groups |  |
| Outcome | Overall survival |  |
| Others | Language: English, German | Grey literature |
