# Supplementary material for: Systematic assessment of the influence of quality of studies on mistletoe in cancer care on the results of a meta-analysis on overall survival
Source: J Cancer Res Clin Oncol. 2024 Apr 29;150(4):219. doi: 10.1007/s00432-024-05742-1 (PMC11056339; doi:10.1007/s00432-024-05742-1)
Supplement: Supplementary file 2 — Supplementary file2 (DOCX 16 KB) [file 432_2024_5742_MOESM2_ESM.docx]

Supplementary file corresponding to the article:

# Systematic assessment of the influence of quality of studies on mistletoe in cancer care on the results of a meta-analysis on overall survival

Jorina Hofinger, University of Jena, Klinik für Innere Medizin II, Jena, Germany.

[jorina.hofinger@outlook.de](mailto:jorina.hofinger@outlook.de), corresponding author, ORCID-ID 0009-0007-7169-3915

Lukas Kaesmann, Jens Buentzel, Martin Scharpenberg, Jutta Huebner

e-supplementary Table 2: Search strategy per database

| Database | Search string (04/22-08/22) |
| --- | --- |
| Medline/Embase via Ovid | 1. mistletoe$.mp. or exp Mistletoe/ or viscum album.mp. or exp Viscum album/ or (“ABNOBAViscum” or “Lektinol” or “Plenosol” or “Isorel” or “Iscucin” or “Iscador” or “Iscar” or “Helixor” or “Eurixor” or “Vysorel”).mp  2. exp neoplasms/ or neoplasm$.mp. or cancer$.mp. or tumo?r$.mp. or malignan$.mp. or oncolog$.mp. or carcinom$.mp. or leuk?emia.mp. or lymphoma.mp. or sarcom$.mp  3. 1 and 2  4. limit 3 to english or limit 3 to german |
| CENTRAL | #1. [mh mistletoe] or mistletoe? or “Viscum album” or “Viscum” or “ABNOBAViscum” or “Lektinol” or “Plenosol” or “Isorel” or “Iscucin” or “Iscador” or “Iscar” or “Helixor” or “Eurixor” or “Vysorel”  #2. [mh neoplasms] or neoplasm* or cancer? or tum*r? or malignan* or oncolog* or carcinom* or leuk*mia or “lymphoma” or sarcoma?  #3. #1 and #2 |
| CINAHL | S1. (MH “Mistletoe” OR TX Mistletoe OR TX “Viscum album” OR TX “Viscum” OR TX “ABNOBAViscum” OR TX “Lektinol” OR TX “Plenosol” OR TX “Isorel” OR TX “Iscucin” OR TX “Iscador” OR TX “Iscar” OR TX “Helixor” OR TX “Eurixor” OR TX “Vysorel”)  S2. MH “Neoplasms+” OR TX neoplasm* OR TX cancer OR TX tumo#r OR TX malignan* OR TX oncolog* OR TX carcinom* OR TX leuk#emia OR TX lymphoma OR TX sarcoma  S3. (LA German OR LA English)  S4. S1 AND S2 AND S3 |
| PsycINFO | S1. (TX Mistletoe OR TX “Viscum album” OR TX “Viscum” OR TX “ABNOBAViscum” OR TX “Lektinol” OR TX “Plenosol” OR TX “Isorel” OR TX “Iscucin” OR TX “Iscador” OR TX “Iscar” OR TX “Helixor” OR TX “Eurixor” OR TX “Vysorel”)  S2. ((DE “Neoplasms” OR DE “Benign Neoplasms” OR DE “Breast Neoplasms” OR DE “Endocrine Neoplasms” OR DE “Leukemias” OR DE “Melanoma” OR DE “Metastasis” OR DE “Nervous System Neoplasms” OR DE “Terminal Cancer”) OR (TX neoplasm* OR TX cancer OR TX tumo#r OR TX malignan* OR DE “oncology” OR TX oncolog* OR TX carcinom* OR TX leuk#emia OR TX lymphoma OR TX sarcoma)  S3. (LA German OR LA English)  S4. S1 AND S2 AND S3 |
| Science Citation Index Expanded (Web of Science) | #1. (TS=Mistletoe* OR TS="Viscum album” OR TS=Viscum OR TS="ABNOBAViscum” OR TS="Lektinol” OR TS="Plenosol” OR TS="Isorel” OR TS="Iscucin” OR TS="Iscador” OR TS="Iscar” OR TS="Helixor” OR TS="Eurixor” OR TS="Vysorel”)  #2. (TS=neoplasm* OR TS=cancer OR TS=cancers OR TS=>tumo$r OR TS=tumo$rs OR TS=malignan* OR TS=oncolog* OR TS=carcinom* OR TS=leuk$emia OR TS=lymphoma OR TS=sarcoma OR TS=sarcomas)  #3. #1 AND #2 |

see Freuding et al. 2019^1^

1. Freuding M, Keinki C, Micke O, Buentzel J, Huebner J. Mistletoe in oncological treatment: a systematic review: Part 1: survival and safety. J Cancer Res Clin Oncol. 2019;145(3):695-707.
