# Supplementary material for: Systematic assessment of the influence of quality of studies on mistletoe in cancer care on the results of a meta-analysis on overall survival
Source: J Cancer Res Clin Oncol. 2024 Apr 29;150(4):219. doi: 10.1007/s00432-024-05742-1 (PMC11056339; doi:10.1007/s00432-024-05742-1)
Supplement: Supplementary file 3 — Supplementary file3 (DOCX 62 KB) [file 432_2024_5742_MOESM3_ESM.docx]

Supplementary file corresponding to the article:

# Systematic assessment of the influence of quality of studies on mistletoe in cancer care on the results of a meta-analysis on overall survival

Jorina Hofinger, University of Jena, Klinik für Innere Medizin II, Jena, Germany.

[jorina.hofinger@outlook.de](mailto:jorina.hofinger@outlook.de), corresponding author, ORCID-ID 0009-0007-7169-3915

Lukas Kaesmann, Jens Buentzel, Martin Scharpenberg, Jutta Huebner

## Excluded studies

43 publications had to be excluded after full text screening. The largest group formed 22 publications whose endpoint was not overall survival ^1-22^. Furthermore, six publications reported on feasibility studies ^23-28^. One study identified via literature lists of other meta-analyses ^29^ turned out to be grey literature and was therefore excluded ^30^. Eight publications were duplicates of studies included into this meta-analysis: Bock et al. ^31^ reported on the same dataset as Schmidt et al. ^32^. The study of Fellmer et al. on gynecological cancer was published thrice ^33-35^. Only the most detailed version (Fellmer 1966)^35^ was included. Likewise, the publications of ^36-38^ were excluded as a more detailed publication of the studies was already included. Last, Grossarth-Maticek et al. ^39^ was excluded as all strata had already been identified within other publications of the author. Another six publications reporting on overall survival had to be excluded because they did not report survival data in a way eligible for transformation into HR and seHR ^40-45^. For detailed description of studies and strata meeting inclusion criteria but not reporting survival data in a way eligible for quantitative analysis please see **Table 4**.

Supplementary table 3: Results of excluded studies and strata

| **Author** | **Year** | **Strata**  **excluded/total*** | **Cancer** | **Detailed reasons for exlusion and results of excluded strata** |
| --- | --- | --- | --- | --- |
| Bar-Sela^40^ | 2013 | 3/3 | Lung | Only median survival times given. Median survival stage III intervention/control: 15.9/13.3 months, stage IV intervention/control: 8.3/8.9 months. Not significant. |
| Dold^46^ | 1991 | 3/4 | Lung | No patient numbers available for some strata. Median survival intervention worse than control group within all strata. |
| Fellmer^35^ | 1966 | 6/8 | Gynecological | Kaplan Meier Curve given for intervention group only. Not comparable. |
| Fritz^47^ | 2018 | 13/15 | Breast | Raw data analysis with worse outcome (p=0.02) for intervention group patients. Most strata reported CI and p values wirthout an effect measure. |
| Grossarth-Maticek Corpus^48^ | 2008 | 6/12 | Gynecological | Six strata only reported median survival differences with CI and p, significant improval of survival under intervention in all but one stratum (cancer of endometrium with strict matching). |
| Grossarth-Maticek Ovar^49^ | 2007 | 6/11 | Gynecological | Six strata only reported median survival differences with CI and p, significant improval of survival under intervention "for nearly all individual studies". |
| Grossarth-Maticek Cervix^50^ | 2007 | 6/10 | Gynecological | Six strata only reported median survival differences with CI and p, significant improval of survival under intervention in all strata. |
| Grossarth-Maticek (Prospective)^51^ | 2006a | 2/4 | Breast | Two strata reported on tumor progression (time to local recurrences, lymphatic or distant metastases), instead of overall survival. "In most cases" significant in favor of intervention group. |
| Grossarth-Maticek (Rand…)^52^ | 2006b | 6/10 | Breast | Six strata only reported median survival differences with CI and p. Positive trends or significant improval under intervention in all strata. |
| Grossarth-Maticek^53^ | 2001 | 7/14 | Multiple | One stratum only reported median survival in favour of intervention, four strata already included into analysis through other publications, two not reporting any survival data. |
| Günczler^54^ | 1969 | 6/9 | Multiple | Two strata reported on five year survival, in favour of intervention group, not significant. Four strata already included into analysis through other publications. |
| Günczler^55^ | 1968 | 2/6 | Gastrointestinal | Two strata only reporting median survival, in favour of intervention group, not significant. |
| Hassauer^56^ | 1979 | 2/6 | Gynecological | Only mean survival of intervention group given for two strata. Not comparable. |
| Hoffmann^57^ | 1979 | 6/8 | Multiple | Six strata only reported mean survival times, no statistical analysis. Statement that there was a better survival in the intervention group. |
| Leroi^42^ | 1982 | 5/5 | Gynecological | No improvement of median survival and five- year- survival within the intervention group. No p-values reported. |
| Lero^41^ | 1979 | 7/7 | Gastrointestinal | Statement that there was a significant improvement of median survival for the intervention group. No p-values reported. No statistical analysis within the strata |
| Leroi^58^ | 1977 | 1/3 | Breast | No survival data given for full study population, subgroups included. |
| Leroi^59^ | 1975 | 5/6 | Multiple | Three-, five- and ten- year- survival only given for intervention group. Not comparable. |
| Majewski^43^ | 1963 | 4/4 | Multiple | Two- year survival presented. Positive trend for patients with endometrium and breast cancer, no survival difference for patients with cervical and and ovarian cancer. No statistical analysis. |
| Salzer^60^ | 1983 | 1/3 | Gastrointestinal | No survival data given for full population, subgroups included. |
| Schreiber^44^ | 1984 | 17/17 | Gynecological | Survival data either given for all patients together, or intervention group only, comparison of observed vs expected deaths. No results intervention vs. control group. |
| Seifert^45^ | 2022 | 2/2 | Multiple | No survival data reported, statement that there was no survival benefit for the intervention group. |

*number of strata excluded/number of all strata in the publication, strata= data strands identified within publications with individual patient and survival data

1. Baek JH, Jeon Y, Han KW, et al.2021 Effect of mistletoe extract on tumor response in neoadjuvant chemoradiotherapy for rectal cancer: a cohort study. World Journal of Surgical Oncology.19(1):178(110).<https://dx.doi.org/10.1186/s12957-021-02293-4>

2. Beuth J, Schneider B, Schierholz J.2008 Impact of complementary treatment of breast cancer patients with standardized mistletoe extract during aftercare: a controlled multicenter comparative epidemiological cohort study. Anticancer research.28(1B):523-527

3. Borrelli E.2001 Evaluation of the quality of life in breast cancer patients undergoing lectin standardized mistletoe therapy. Minerva Medica.92(Suppl 1 Nr 3):105-107

4. Büssing A, Bückner U, Enser-Weis U, et al.2008 Abstract: Modulation of chemotherapy-associated immunosuppression by intravenous application of Viscum album L. Extract (Iscador): A randomised phase II study. European Journal of Integrative Medicine.1:2-3.<https://doi.org/10.1016/j.eujim.2008.08.084>

5. Enesel MB, Acalovschi I, Grosu V, et al.2005 Perioperative application of the Viscum album extract Isorel in digestive tract cancer patients. Anticancer Research.25(6C):4583-4590

6. Friedel We MHBPRZKS. 2009 Systematic evaluation of the clinical effects of supportive mistletoe treatment within chemo-and/or radiotherapy protocols and long-term mistletoe application in ….

7. Heiny B.1991 Additive Therapie mit standardisiertem Mistelextrakt reduziert die Leukopenie und verbessert die Lebensqualität von Patientinnen mit fortgeschrittenem Mammakarzinom unter palliativer Chemotherapie (VEC-schema). Krebsmedizin.12:1-14

8. Heiny BM, Albrecht V, Beuth J.1998 Lebensqualitätsstabilisierung durch Mistellektin-1 normierten Extrakt beim fortgeschrittenen kolorektalen Karzinom. Der Onkologe.4(S1):35-39

9. Jeon Y, Baek JH, Han KW, et al.2022 The impact of mistletoe extract on tumor regression in neoadjuvant chemoradiotherapy for rectal cancer. European Journal of Surgical Oncology.48(2):e100.<https://dx.doi.org/10.1016/j.ejso.2021.12.152>

10. Kaiser G, Büschel M, Horneber M, et al. 2001 Studiendesign und erste Ergebnisse einer prospektiven placebokontrollierten, randomisierten Studie mit AbnobaViscum Mali 4. In: Scheer R BR, Becker H, Berg PA, editor. Die Mistel in der Tumortherapie: Grundlagenforschung und Klinik Essen: KVC. p. 485-505.

11. Kim K, Yook J, Eisenbraun J, et al.2012 Quality of life, immunomodulation and safety of adjuvant mistletoe treatment in patients with gastric carcinoma - a randomized, controlled pilot study. . BMC Complement Altern Med.12(172):1472- 1479

12. Lenartz D, Stoffel B, Menzel J, et al.1996 Immunoprotective activity of the galactoside-specific lectin from mistletoe after tumor destructive therapy in glioma patients. Anticancer Research.16(6B):3799-3802

13. Longhi A, Reif M, Mariani E, et al.2014 A Randomized Study on Postrelapse Disease-Free Survival with Adjuvant Mistletoe versus Oral Etoposide in Osteosarcoma Patients. Evidence-based complementary and alternative medicine : eCAM.2014:210198, 210199 pages.<https://doi.org/10.1155/2014/210198>

14. Oei SL, Thronicke A, Kröz M, et al.2020 Impact of Oncological Therapy and Viscum album L Treatment on Cancer-Related Fatigue and Internal Coherence in Nonmetastasized Breast Cancer Patients. Integrative Cancer Therapies.19:1-16.<https://doi.org/10.1177/1534735420917211>

15. Pelzer F, Tröger W.2018 Complementary Treatment with Mistletoe Extracts During Chemotherapy: Safety, Neutropenia, Fever, and Quality of Life Assessed in a Randomized Study. Journal of Alternative & Complementary Medicine.24(9/10):954-961.<https://doi.org/10.1089/acm.2018.0159>

16. Piao BK WY, Xie GR, Mansmann U, Matthes H, Beuth J, et al.2004 Impact of complementary mistletoe extract treatment on quality of life in breast, ovarian and non-small cell lung cancer patients. A prospective randomized controlled clinical trial. . Anticancer Res.24(1):303-309

17. Schumacher K, Schneider B, Reich G, et al.2003 Influence of postoperative complementary treatment with lectin-standardized mistletoe extract on breast cancer patients. A controlled epidemiological multicentric retrolective cohort study. Anticancer research.23(6D):5081-5087

18. Semiglasov VF, Stepula VV, Dudov A, et al.2004 The standardised mistletoe extract PS76A2 improves QoL in patients with breast cancer receiving adjuvant CMF chemotherapy: a randomised, placebo-controlled, double-blind, multicentre clinical trial. Anticancer Research.24(2C):1293-1302

19. Semiglazov VF, Stepula VV, Dudov A, et al.2006 Quality of life is improved in breast cancer patients by standardised mistletoe extract PS76A2 during chemotherapy and follow-up: a randomised, placebo-controlled, double-blind, multicentre clinical trial. . Anticancer Res.26(2B):1519-1529

20. Steuer-Vogt MK, Bonkowsky V, Scholz M, et al.2006 Einfluss eines ML-1-normierten Mistelextraktes auf die Lebensqualität bei Patienten mit Kopf-Hals-Karzinomen. HNO.54(4):277-286.<https://doi.org/10.1007/s00106-005-1318-y>

21. Tröger W, Zdrale Z, Tišma N, et al.2014 Additional Therapy with a Mistletoe Product during Adjuvant Chemotherapy of Breast Cancer Patients Improves Quality of Life: An Open Randomized Clinical Pilot Trial. Evidence-based complementary and alternative medicine : eCAM.2014:430518.<https://doi.org/10.1155/2014/430518>

22. Zaenker KS.2012 A Specific Mistletoe Preparation (Iscador-Qu®) in Colorectal Cancer (CRC) Patients: More than Just Supportive Care? J Cancer Sci Ther.4(9):264-270.<https://doi.org/10.4172/1948-5956.1000153>

23. Huntley A, Duncan L, Feder G, et al.2020 A Feasibility Study of the Mistletoe and Breast Cancer (MAB) Trial: A Protocol for a Randomised Double-blind Controlled Trial. BMC Pilot Studies.<https://doi.org/10.21203/rs.3.rs-50668/v1>

24. Huntley A, Duncan L, Feder G, et al.2021 Reflections on the first UK mistletoe clinical trial: a feasibility, placebo -controlled randomised trial. European Journal of Integrative Medicine.48:101930.<https://dx.doi.org/10.1016/j.eujim.2021.101930>

25. Loewe-Mesch A, Kuehn JJ, Borho K, et al.2008 Adjuvante simultane Mistel-/Chemotherapie bei Mammakarzinom - Einfluss auf Immunparameter, Lebensqualität und Verträglichkeit. Forschende Komplementärmedizin 15(1):22-30.<https://doi.org/10.1159/000112860>

26. Longhi A, Mariani E, Kuehn JJ.2009 A randomized study with adjuvant mistletoe versus oral Etoposide on post relapse disease-free survival in osteosarcoma patients. European Journal of Integrative Medicine.1:27-33.<https://doi.org/10.1016/j.eujim.2009.02.005>

27. Tröger W, Galun D, Reif M, et al.2014 Quality of Life of Patients With Advanced Pancreatic Cancer During Treatment With Mistletoe. Deutsches Ärzteblatt International.111(29-30):493-502.<https://doi.org/10.3238/arztebl.2014.0493>

28. Tröger W, Jezdić S, Zdrale Z, et al.2009 Quality of life and neutropenia in patients with early stage breast cancer: a randomized pilot study comparing additional treatment with mistletoe extract to chemotherapy alone. Breast Cancer: Basic and Clinical Research.3(1):35-45.<https://doi.org/10.4137/BCBCR.S2905>

29. Loef M, Walach H.2020 Quality of life in cancer patients treated with mistletoe: a systematic review and meta-analysis. BMC Complementary Medicine and Therapies.20(1):227.<https://doi.org/10.1186/s12906-020-03013-3>

30. Grah C. 2010 Misteltherapie bei nichtkleinzelligem Bronchialkarzinom: Randomisierte, offene Phase-II-Studie zur Untersuchung der Verträglichkeit, Sicherheit und Wirksamkeit von ….

31. Bock PR, Friedel WE, Hanisch J, et al.2004 Retrolective, comparative, epidemiological cohort study with parallel groups design for evaluation of efficacy and safety of drugs with "well-established use". Forschende Komplementarmedizin und klassische Naturheilkunde.11 Suppl 1(1):23-29.<https://doi.org/10.1159/000080572>

32. Schmidt N, Edgar W. Die postoperative komplementäre Therapie des primären Mammakarzinom mit lektinnormiertem Mistelextrakt: eine epidemiologische, kontrollierte retrolektive Kohortenstudie. Universitätsmedizin Berlin2007.

33. Fellmer K.1967 Nachbehandlung bestrahlter Genitalkarzinome mit dem Viscum-album-Präparat Iscador® zur Rezidivprophylaxe. Medizinische Klinik.62:305-307

34. Fellmer KE.1968 A clinical trial of Iscador. British Homeopathic Journal.57(01):43-47

35. Fellmer KE, Fellmer C.1966 Nachbehandlung bestrahlter Genitalkarzinome mit dem Viscum-album-Präparat" Iscador. Krebsarzt.21(3):174-185

36. Günczler M, Osika C, Salzer G.1968 Ergebnisse von Resektion und Nachbehandlung beim Magenkarzinom. Wiener Klinische Wochenzeitschrift.80(6):105-106

37. Salzer G.1975 Klinischer Versuch zur Verbesserung des Schicksals "Radikaloperierter" Bronchuskarzinom-Patienten. Zeitschrift für Erkrankungen der Atmungsorgane.142:127-131

38. Salzer G, Havelec L.1978 Rezidivprophylaxe bei operierten Bronchuskarzinompatienten mit dem Mistelpräparat Iscador® Ergebnisse eines klinischen Versuchs aus den Jahren 1969-1971. Onkologie.1(6):264-267.<https://doi.org/10.1159/000213966>

39. Grossarth-Maticek R, Kiene H, Baumgartner S, et al.2001 Verlängerung der Überlebenszeit von Krebspatienten unter Misteltherapie (Iscador). Swiss Journal of Integrative Medicine.13:217-225

40. Bar-Sela G, Wollner M, Hammer L, et al.2013 Mistletoe as complementary treatment in patients with advanced non-small-cell lung cancer treated with carboplatin-based combinations: a randomised phase II study. European Journal of Cancer 49(5):1058-1064.<https://doi.org/10.1016/j.ejca.2012.11.007>

41. Leroi R.1979 Die Iscadorbehandlung bei inoperablen kolo-rektalen Tumoren. Krebsgeschehen.11(6):163-165

42. Leroi R, Hajto T.1982 Die Iscadortherapie beim Ovarialkarzinom. Krebsgeschehen.14(2):38-44

43. Majewski A, Bentele W.1963 Über Zusatzbehandlung beim weiblichen Genitalkarzinom. Zentralblatt für Gynäkologie.85(20):696-700

44. Schreiber K, Stumpf C.1984 Iscador in der postoperativen Therapie des Ovarialkarzinoms: Ergebnisse 24jähriger Therapie. Erfahrungsheilkunde.33(6):349-358

45. Seifert G, Blakeslee SB, Calaminus G, et al.2022 Integrative medicine during the intensive phase of chemotherapy in pediatric oncology in Germany: a randomized controlled trial with 5-year follow up. BMC Cancer.22(1):652.<https://doi.org/10.1186/s12885-022-09703-0>

46. Dold U, Edler L, Mäurer HC, et al. 1991 Krebszusatztherapie beim fortgeschrittenen nicht-kleinzelligen Bronchialkarzinom: multizentrische kontrollierte Studie zur Prüfung der Wirksamkeit von Iscador und Polyerga. Stuttgart: Georg Thieme Verlag. 139 p

47. Fritz P, Dippon J, Müller S, et al.2018 Is Mistletoe Treatment Beneficial in Invasive Breast Cancer? A New Approach to an Unresolved Problem. Anticancer Research.38(3):1585-1593

48. Grossarth-Maticek R, Ziegler R.2008 Randomized and non-randomized prospective controlled cohort studies in matched pair design for the long-term therapy of corpus uteri cancer patients with a mistletoe preparation (Iscador). Eur J Med Res.13(3):107-120

49. Grossarth-Maticek R, Ziegler R.2007 Prospective controlled cohort studies on long-term therapy of ovarian cancer patients with mistletoe (Viscum album L.) extracts (Iscador). Arzneimittelforschung.57(10):665-678.<https://doi.org/10.1055/s-0031-1296666>

50. Grossarth-Maticek R, Ziegler R.2007 Prospective controlled cohort studies on long-term therapy of cervical cancer patients with a mistletoe preparation (Iscador). Forschende Komplementärmedizin.14(3):140-147.<https://doi.org/10.1159/000102956>

51. Grossarth-Maticek R, Ziegler R.2006 Prospective controlled cohort studies on long-term therapy of breast cancer patients with a mistletoe preparation (Iscador). Forschende Komplementarmedizin 13(5):285-292.<https://doi.org/10.1159/000095378>

52. Grossarth-Maticek R, Ziegler R.2006 Randomised and non-randomised prospective controlled cohort studies in matched-pair design for the long-term therapy of breast cancer patients with a mistletoe preparation (Iscador): a re-analysis. Eur J Med Res.11:485-495

53. Grossarth-Maticek R, Kiene H, Baumgartner SM, et al.2001 Use of Iscador, an extract of European mistletoe (Viscum album), in cancer treatment: prospective nonrandomized and randomized matched-pair studies nested within a cohort study. . Altern Ther Health Med.7(3):57-78

54. Günczler M, Salzer G.1969 Iscador-Therapie in der Nachbehandlung operierter Carcinome. Österreichische Ärztezeitung.24(20):2290-2294

55. Günczler M.1968 Ergebnisse und Erfahrungen in der Krebstherapie mit Iscador®. I. Das Magencarcinom. Beitrag Erweiterte Heilkunde.21(6):188-195

56. Hassauer W, Gutsch J, Burkhardt R.1979 Welche Erfolgsaussichten bietet die Iscador®-Therapie beim fortgeschrittenen Ovarialkarzinom? Oncology Research and Treatment.2(1):28-36

57. Hoffmann J.1979 Die Iscador-Behandlung bei Lebermetastasen. Krebsgeschehen.1979:172-175

58. Leroi R.1977 Nachbehandlung des operierten Mammakarzinoms mit Viscum Album. Helvetica chirurgica acta.44(3):403-414

59. Leroi R.1975 Malignomtherapie mit neuen Iscador-Präparaten. Krebsgeschehen.7(5):124-126

60. Salzer G, Havelec L.1983 Adjuvante Iscador-Behandlung nach operiertem Magenkarzinom. Ergebnisse einer randomisierten Studie. . Krebsgeschehen.15(4):106-110
