# Supplementary material for: Systematic assessment of the influence of quality of studies on mistletoe in cancer care on the results of a meta-analysis on overall survival
Source: J Cancer Res Clin Oncol. 2024 Apr 29;150(4):219. doi: 10.1007/s00432-024-05742-1 (PMC11056339; doi:10.1007/s00432-024-05742-1)

Supplementary plots of meta-analysis corresponding to the article:

# Systematic assessment of the influence of quality of studies on mistletoe in cancer care on the results of a meta-analysis on overall survival

Jorina Hofinger, University of Jena, Klinik für Innere Medizin II, Jena, Germany.

[jorina.hofinger@outlook.de](mailto:jorina.hofinger@outlook.de), corresponding author, ORCID-ID 0009-0007-7169-3915

Lukas Kaesmann, Jens Buentzel, Martin Scharpenberg, Jutta Huebner

| **Nr.** | **Title of meta-analysis** | **HR with 95%-CI** | **I²** | **Chi²** | **Tau²** |
| --- | --- | --- | --- | --- | --- |
| 1 | All strata providing HR | 0.61 [0.53;0.71] | 72% | 305.4 [p<0.01] | 0.1202 |
| 2 | All stata describing full populations | 0.63 [0.53;0.74] | 80% | 222.68 [p<0.01] | 0.1448 |
| 3 | Subgroups mistletoe preparation | 0.61 [0.53;0.71] | 72% | 305.4 [p<0.01] | 0.1202 |
|  | 3.1 Iscador | 0.59 [0.52;0.66] | 48% | 162.24[p<0.01] | 0.0748 |
|  | 3.2 Multiple mistletoe preparations | 1.53 [0.99;2.38] | 85% | 6.84 [p<0.01] | 0.0865 |
| 4 | Subgroups cancer Type | 0.61 [0.53;0.71] | 72% | 305.4 [p<0.01] | 0.1202 |
|  | 4.1 Breast cancer | 0.65 [0.47;0.90] | 85% | 126.37 [p<0.01] | 0.1914 |
|  | 4.2 Colorectal cancer | 0.50 [0.36;0.70] | 17% | 2.4 [p=0.30] | 0.0323 |
|  | 4.3 Gastric, pancreatic and liver cancer | 0.60 [0.50;0.71] | 9% | 18.65 [p=0.35] | 0.0232 |
|  | 4.4 Gynecological cancer other than breast | 0.47 [0.40;0.55] | 0% | 18.51 [p=0.62] | 0.0146 |
|  | 4.5 Lung cancer | 0.66 [0.42;1.04] | 45% | 21.92 [p=0.04] | 0.1833 |
|  | 4.6 Multiple cancer types | 0.57 [0.42;0.77] | 64% | 8.42 [p=0.04] | 0.0481 |
|  | 4.7 Skin cancer | 0.80 [0.54;1.19] | 60% | 15.11[p=0.02] | 0.1062 |
| 5/6 | Subgroups study quality | 0.61 [0.53;0.71] | 72% | 305.4 [p<0.01] | 0.1202 |
|  | 5.1 Nonrandomized, without peer review (PR) | 0.56 [0.44;0.72] | 39% | 35.79 [p=0.03] | 0.0790 |
|  | 5.2 Nonrandomized, with PR | 0.57 [0.45;0.71] | 83% | 202.17 [p<0.01] | 0.1519 |
|  | 6.1 Randomized, without PR | 0.80 [0.69;0.94] | 0% | 5.03 [p=0.75] | <0.0001 |
|  | 6.2 Randomized, with PR | 0.61 [0.45;0.83] | 66% | 53.46 [p<0.01] | 0.1347 |
| 7 | Subgroups risk of bias (randomized) | 0.67 [0.54;0.82] | 59% | 65.68 [p<0.01] | 0.0885 |
|  | 7.1 High risk of bias | 0.66 [0.55;0.80] | 1% | 19.26 [p=0.44] | 0.0360 |
|  | 7.2 Some risk of bias | 0.78 [0.30;2.00] | 84% | 45.09 [p<0.01] | 0.4569 |
| 8 | Subgroups risk of bias (nonrandomized) | 0.57 [0.48;0.67] | 75% | 239.12 [p<0.01] | 0.1193 |
|  | 8.1 Critical risk of bias | 0.50 [0.39;0.66] | 21% | 2.53 [p=0.28] | 0.0158 |
|  | 8.2 Serious risk of bias | 0.61 [0.50;0.73] | 78% | 216.3 [p<0.01] | 0.1293 |
|  | 8.3 Moderate risk of bias | 0.35 [0.24;0.50] | 0% | 2.41 [p=0.88] | <0.0001 |
|  | 8.4 No information on risk of bias | 0.46 [0.26;0.81] | 0% | 0.01 [p=0.93] | <0.0001 |
| 9 | Prospective studies only | 0.62 [0.52;0.72] | 52% | 115.13 [p<0.01] | 0.0863 |
|  | 9.1 Intervention = suggestion of mistletoe therapy | 0.56 [0.45;0.69] | 0% | 7.64 [p=0.75] | <0.0001 |
|  | 9.2 Interventional studies | 0.74 [0.59;0.92] | 59% | 56.15 [p<0.01] | 0.0778 |
|  | 9.3 Observational studies | 0.50 [0.41;0.60] | 52% | 39.22 [p<0.01] | 0.0428 |
| 10 | Subgroups timeline of study | 0.61 [0.53;0.71] | 72% | 305.4 [p<0.01] | 0.1202 |
|  | 10.1 Prospective case, retrospective control group | 0.78 [0.59;1.02] | 0% | 0.38 [p=0.54] | <0.0001 |
|  | 10.2 Prospective studies | 0.62 [0.52;0.72] | 52% | 115.13 [p<0.01] | 0.0863 |
|  | 10.3 Retrolective studies | 0.56 [0.47;0.68] | 0% | 2.59 [p=0.86] | <0.0001 |
|  | 10.4 Retrospective studies | 0.57 [0.32; 1.01] | 89% | 128.77 [p<0.01] | 0.3893 |
|  | 10.5 Retrospective formation of control group | 0.56 [0.32;0.95] | 66% | 20.78 [p<0.01] | 0.1430 |
| 11 | Treatment of control group | 0.61 [0.53;0.71] | 72% | 305.4 [p<0.01] | 0.1202 |
|  | 10.1 Chemotherapy | 0.45 [0.33;0.60] | 0% | 1.01 [p=0.91] | <0.0001 |
|  | 10.2 Insufficient mistletoe therapy | 0.53 [0.37;0.74] | 45% | 1.83 [p=0.18] | 0.0294 |
|  | 10.3 No special treatment of the control group | 0.62 [0.53;0.74] | 74% | 245.78 [p<0.01] | 0.1351 |
|  | 10.4 Control group treated with complementary methods other than mistletoe | 0.49 [0.40;0.61] | 28% | 18.14 [p=0.15] | 0.0421 |
| 12 | All strata giving information on dropouts | 0.57 [0.47;0.69] | 52% | 52.25 [p<0.01] | 0.0931 |
| 13 | All strata with zero dropouts | 0.61 [0.40;0.94] | 0% | 2.59 [p=0.63] | 0 |
| 14 | Early vs. advanced tumor stages | 0.61 [0.52;0.72] | 75% | 275.37 [p<0.01] | 0.1362 |
|  | 14.1 Tumor stage IV | 0.61 [0.51;0.73] | 0% | 6.82 [p=0.74] | <0.0001 |
|  | 14.2 Strata describing stage I-IV | 0.57 [0.44;0.74] | 88% | 196.47 [p<0.01] | 0.2002 |
|  | 14.3 Tumor stage I-III | 0.58 [0.48;0.69] | 52% | 68.54 [p<0.01] | 0.0918 |
| 15 | Early stages (I-III) by risk of bias | 0.58 [0.48;0.69] | 52% | 68.54 [p<0.01] | 0.0918 |
|  | 15.1 Critical risk of bias | 0.50 [0.39;0.66] | 21% | 2.53 [p=0.28] | 0.0158 |
|  | 15.2 High risk of bias | 0.65 [0.52;0.82] | 0% | 5.04 [p=0.75] | 0.0004 |
|  | 15.3 Serious risk of bis | 0.58 [0.48;0.70] | 0% | 11.63 [p=0.48] | 0.0227 |
|  | 15.4 Moderate risk of bias | 0.35 [0.21;0.57] | 0% | 1.26 [p= 0.74] | <0.0001 |
|  | 15.5 Some risk of bias | 0.66 [0.18; 2.41] | 90% | 30.71 [p=0.48] | 0.0227 |
| 16 | Advanced tumors (IV) by risk of bias | 0.61 [0.51;0.73] | 0% | 6.82 [p=0.74] | <0.0001 |
|  | 16.1 High risk of bias | 0.68 [0.38;1.22] | 33% | 4.45 [p=0.22] | 0.1076 |
|  | 16.2 Serious risk of bias | 0.58 [0.46;0.73] | 0% | 1.76 [p=0.88] | 0 |
| 17 | Skin cancer early vs. advanced | 0.85 [0.51;1.40] | 67% | 12.00 [p=0.02] | 0.1434 |
|  | 17.1 Early stages (I-III) | 0.92 [0.47;1.80] | 71% | 10.33 [p=0.02] | 0.2068 |
| 18 | Breast cancer early vs. advanced | 0.63 [0.43;0.93] | 89% | 115.76 [p<0.01] | 0.2347 |
|  | 18.1 Strata describing stage I-IV | 0.67 [0.12;3.66] | 94% | 34.82 [p<0.01] | 1.4699 |
|  | 18.2 Early stages (I-III) | 0.58 [0.46;0.71] | 5% | 9.5 [p=0.39] | 0.0231 |
| 19 | Gynecological cancer early vs. advanced | 0.56 [0.44;0.72] | 83% | 204.16 [p<0.01] | 0.1691 |
|  | 19.1 Advanced tumors (stage IV) | 0.57 [0.46;0.72] | 0% | 4.82 [p=0.68] | <0.0001 |
|  | 19.2 Strata describing stage I-IV | 0.49 [0.29;0.82] | 94% | 165.23 [p<0.01] | 0.3849 |
|  | 19.3 Early stages (I-III) | 0.54 [0.46;0.64] | 0% | 15.88 [p=0.46] | 0.0263 |
| 20 | Gastrointestinal cancer early vs. advanced | 0.53 [0.48;0.59] | 0% | 10.65 [p=0.71] | <0.0001 |
|  | 20.1 Advanced tumors (stage IV) | 0.64 [0.46;0.89] | 0% | 0 [p=0.96] | 0 |
|  | 20.2 Strata describing stage I-IV | 0.52 [0.46;0.59] | 0% | 2.08 [p=0.91] | <0.0001 |
|  | 20.3 Early stages (I-III) | 0.52 [0.34;0.76] | 30% | 7.12 [p=0.21] | 0.0813 |
| 21 | Lung cancer early vs. advanced | 0.70 [0.47;1.03] | 36% | 14.02 [p=0.12] | 0.3166 |
|  | 21.1 Strata describing stage I-IV | 0.85 [0.70;1.02] | 0% | 1.01 [p=0.60] | 0 |
|  | 21.2 Early stages (I-III) | 0.54 [0.23;1.26] | 43% | 8.72 [p=0.12] | 0.3166 |
| 22 | Subgroups status of HR | 0.61 [0.53;0.71] | 72% | 305.4 [p<0.01] | 0.1202 |
|  | 22.1 HR had to be calculated with the Tierney-method | 0.68 [0.56;0.83] | 75% | 173.23 [p<0.01] | 0.1207 |
|  | 22.2 HR had to be calculated from raw data | 0.44 [0.32;0.59] | 0% | 0.57 [p=0.90] | <0.0001 |
|  | 22.3 HR was given | 0.54 [0.44;0.67] | 53% | 81.01 [p<0.01] | 0.0972 |
| 23 | Subgroups timeline of mistletoe therapy | 0.61 [0.53;0.71] | 72% | 305.4 [p<0.01] | 0.1202 |
|  | 23.1 Mistletoe given after conventional therapy | 0.74 [0.62;0.90] | 32% | 36.78 [p=0.06] | 0.0519 |
|  | 23.2 Mistletoe given during adjuvant chemotherapy | 0.55 [0.45;0.67] | 0% | 2.17 [p=0.83] | <0.0001 |
|  | 23.3 Mistletoe during conventional treatment | 0.65 [0.35;1.18] | 73% | 18.2 [p<0.01] | 0.1790 |
|  | 23.4 Long term mistletoe treatment | 0.49 [0.42;0.57] | 30% | 58.98 [p=0.03] | 0.0365 |
|  | 23.5 Timeline of treatment not specified | 0.74 [0.42;1.29] | 94% | 109 [p<0.01] | 0.3182 |

A hazard ratio (HR)< 1 indicates the superiority of the intervention group (mistletoe therapy), while a HR>1 corresponds to the superiority of the control group. P values < 0.05 were considered to indicate statistical significance.


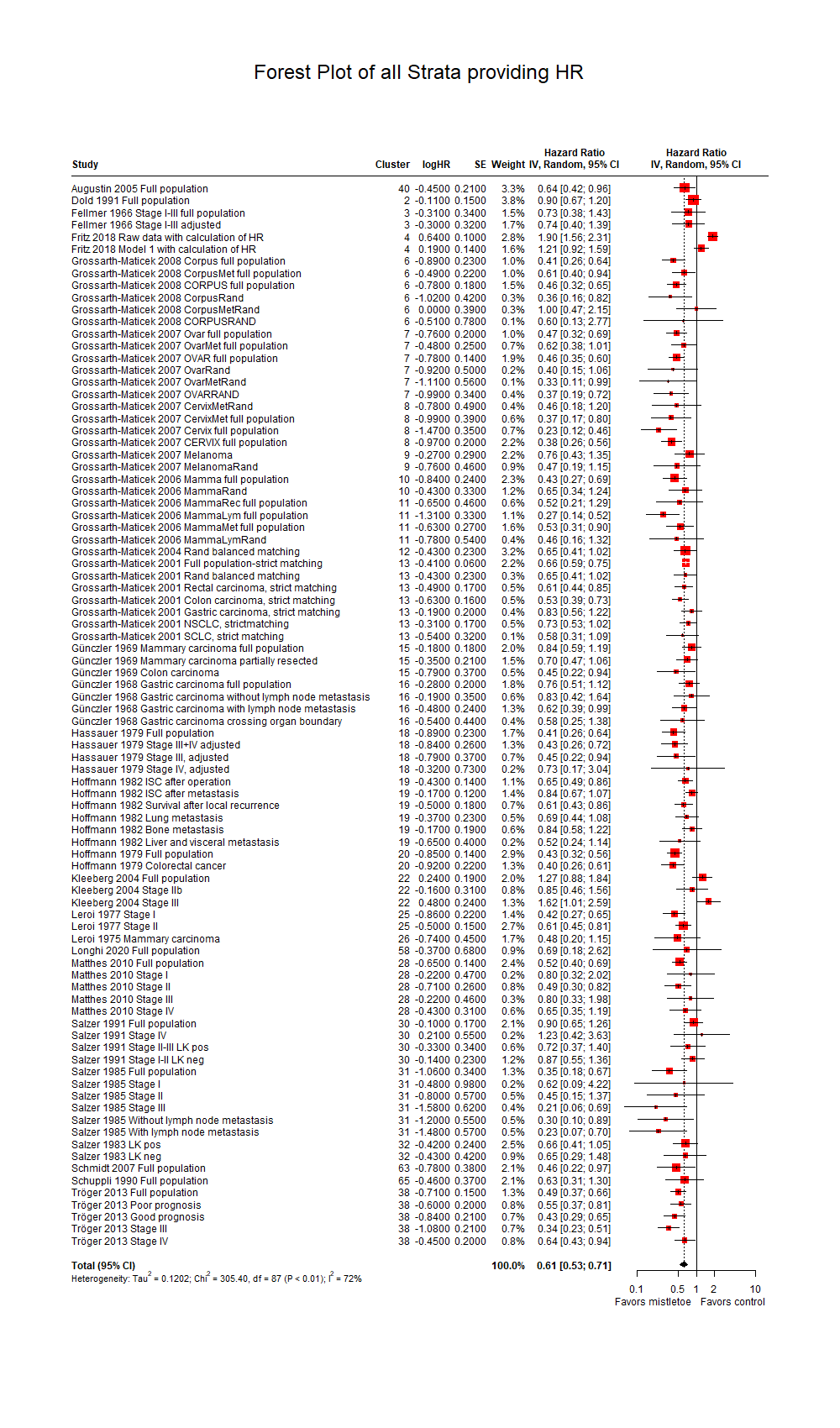

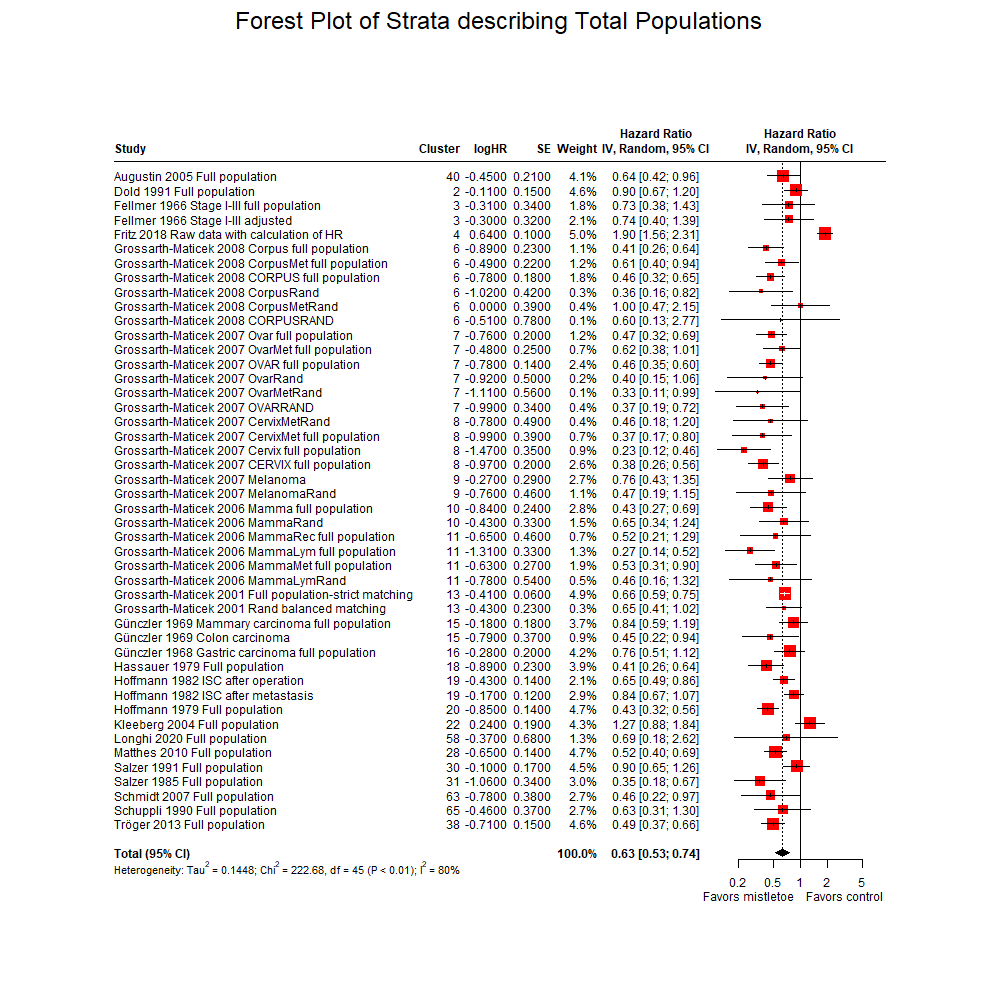

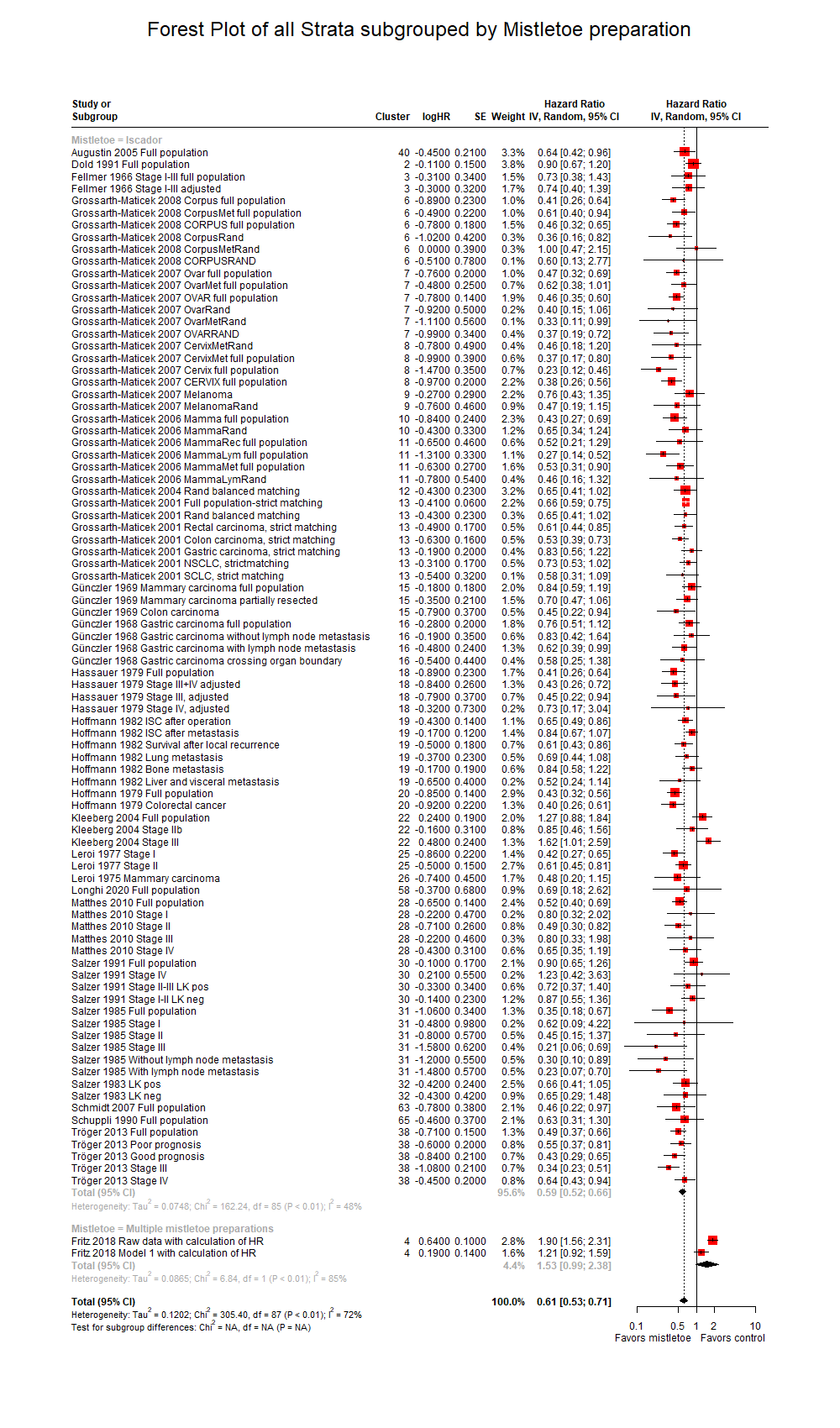

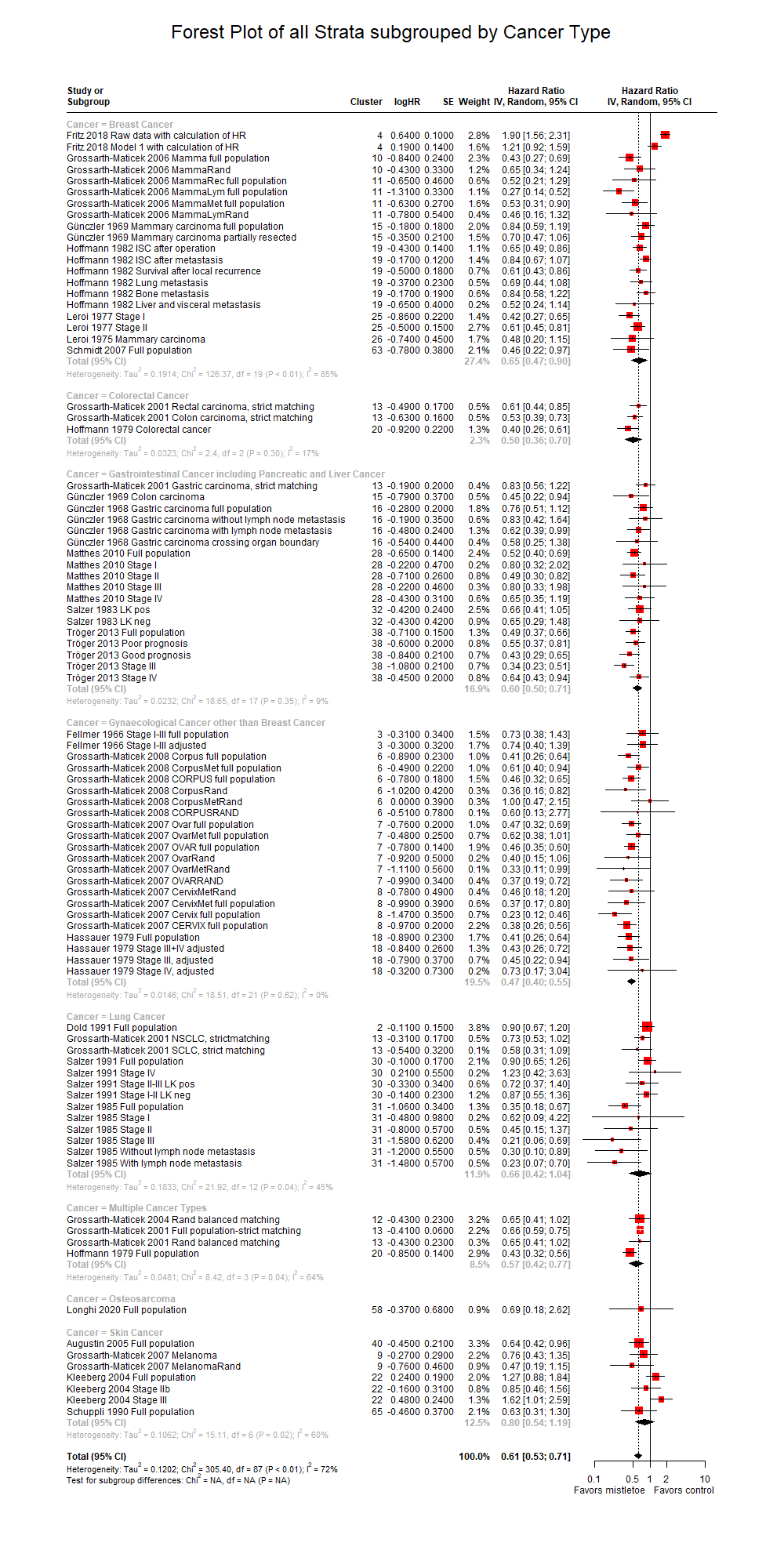

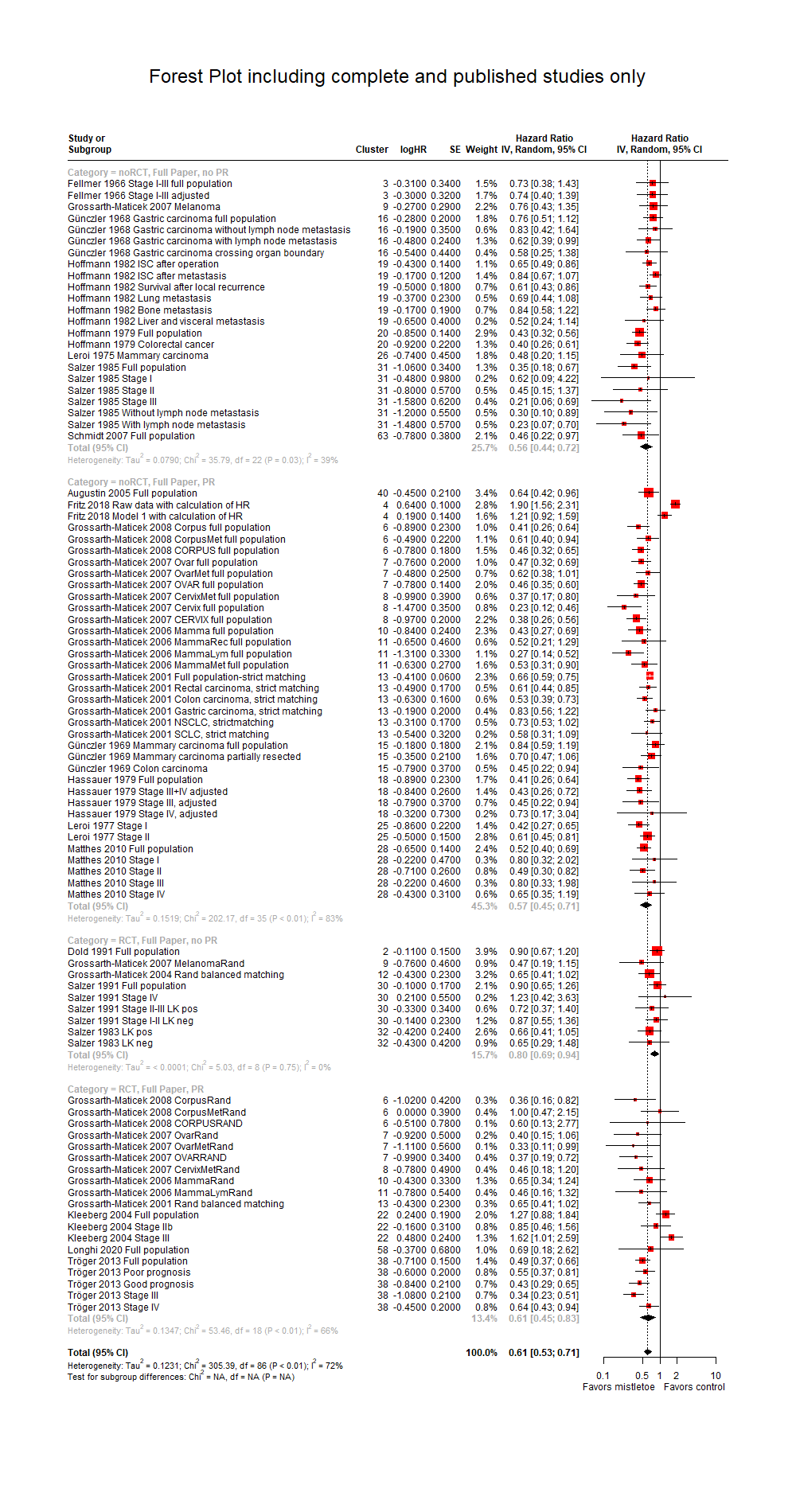

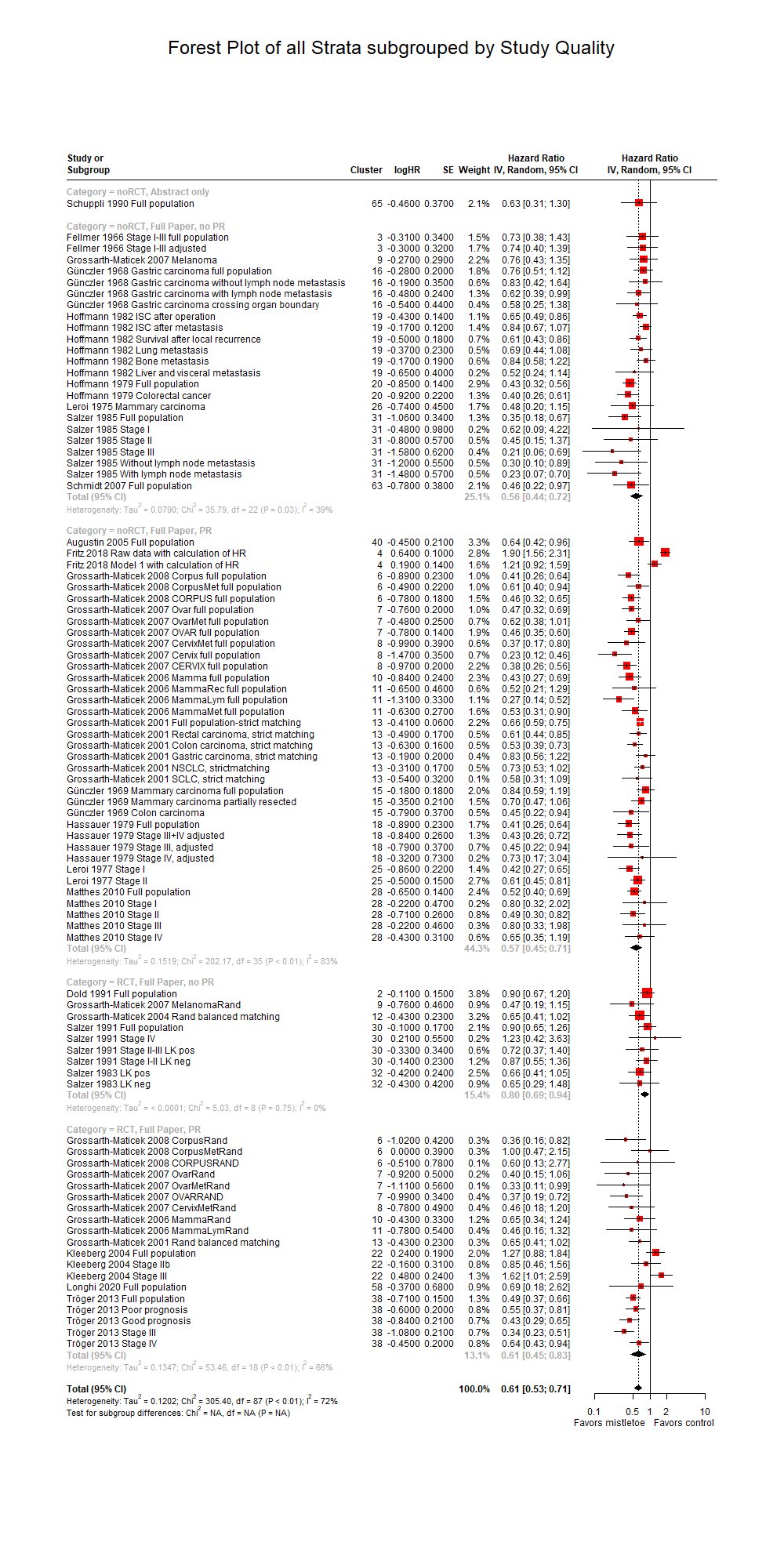


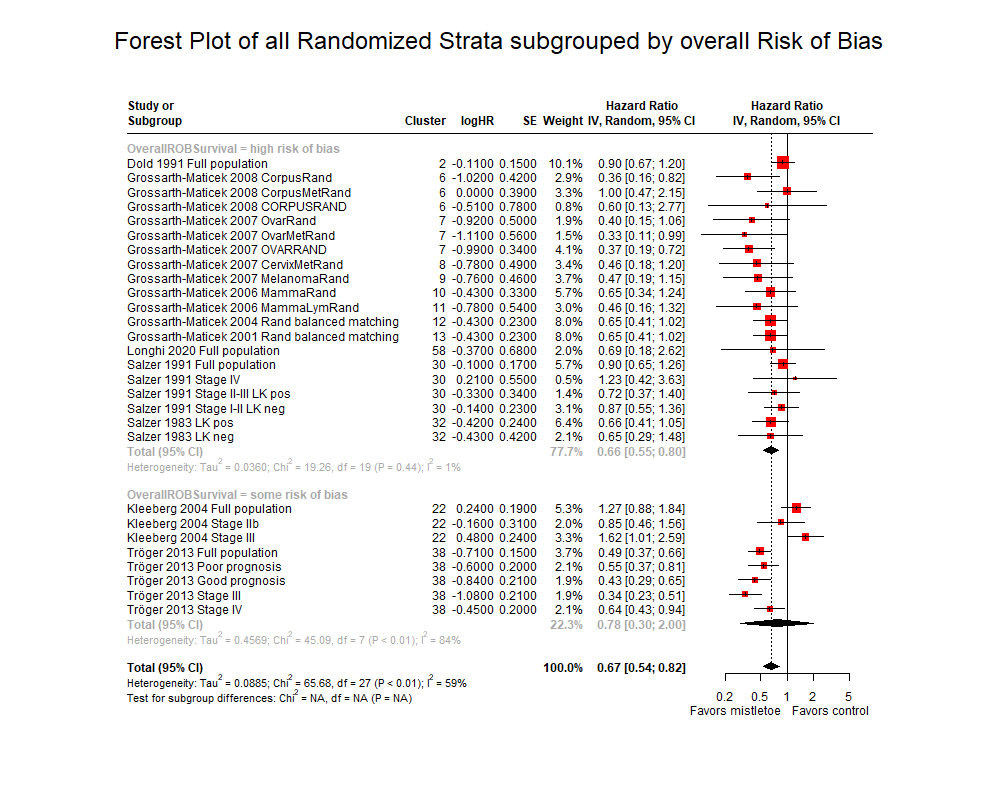


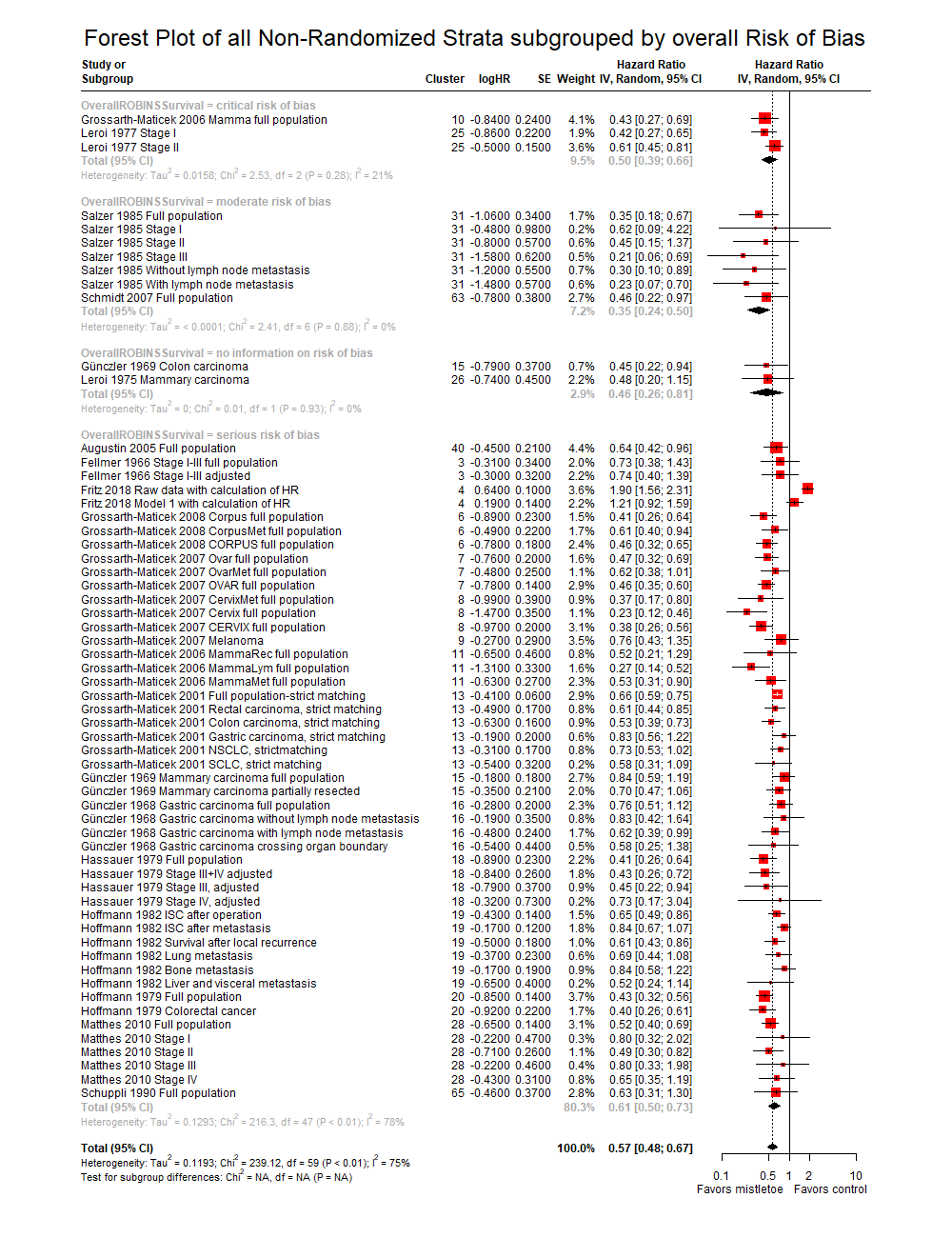


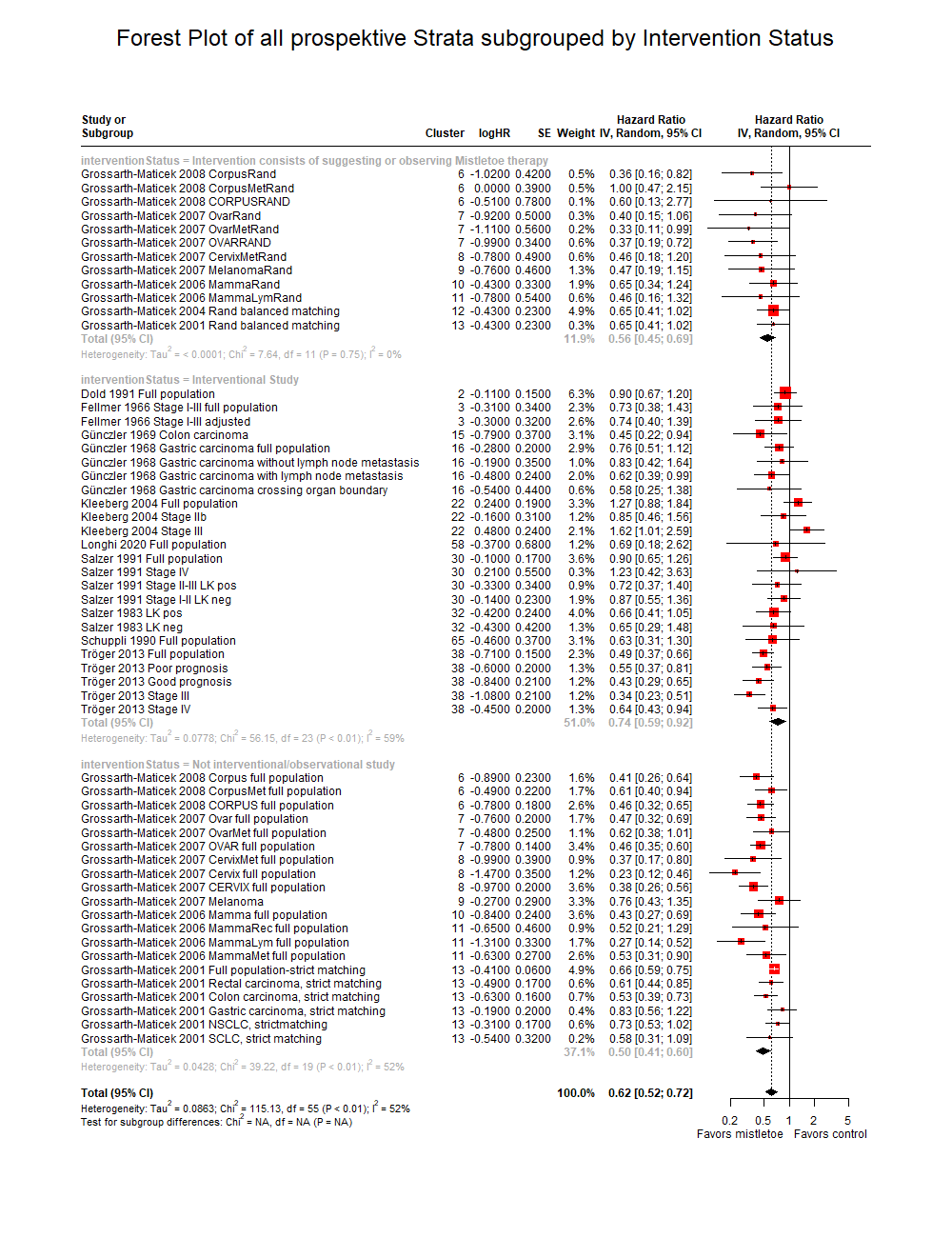

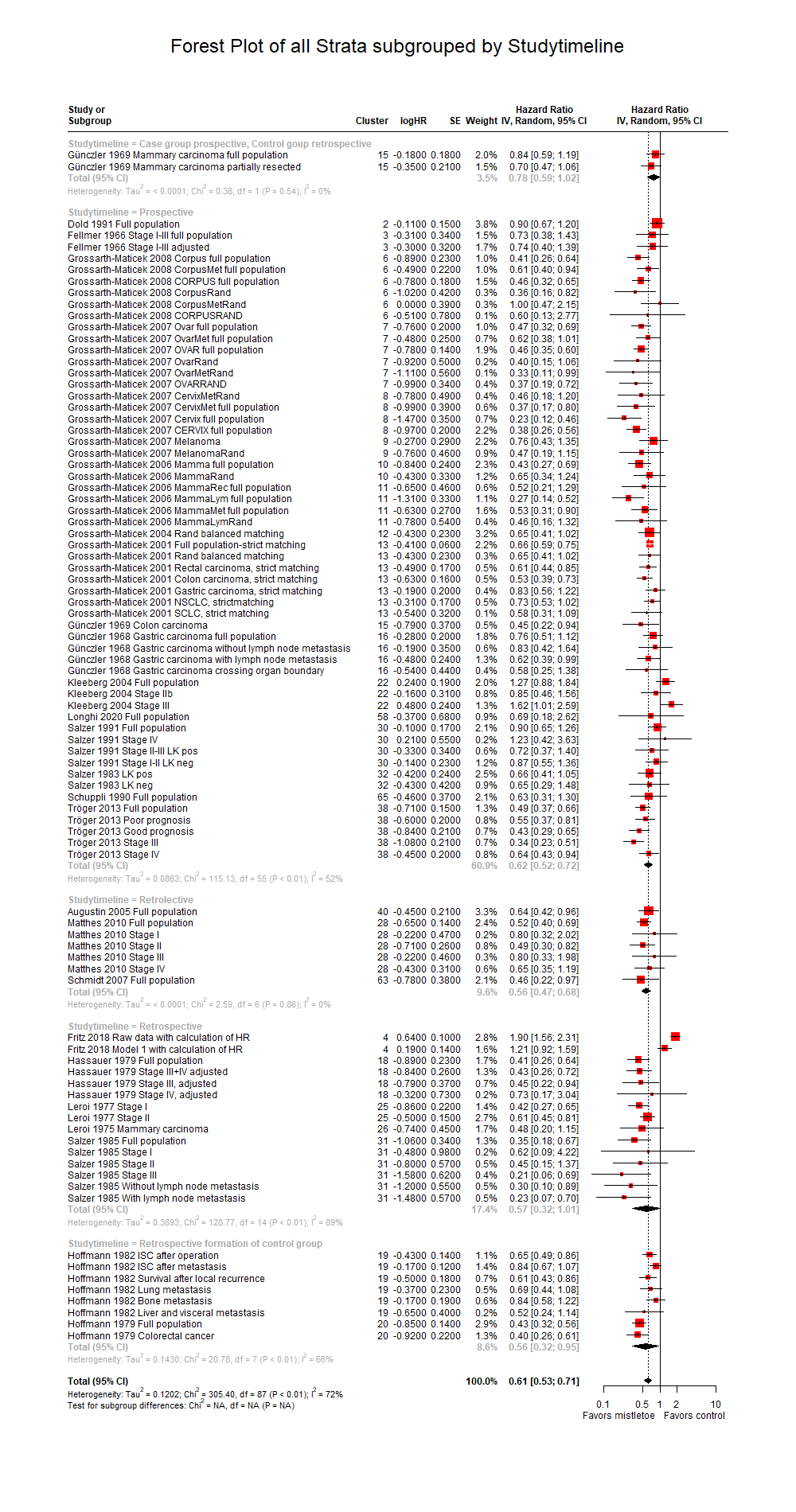


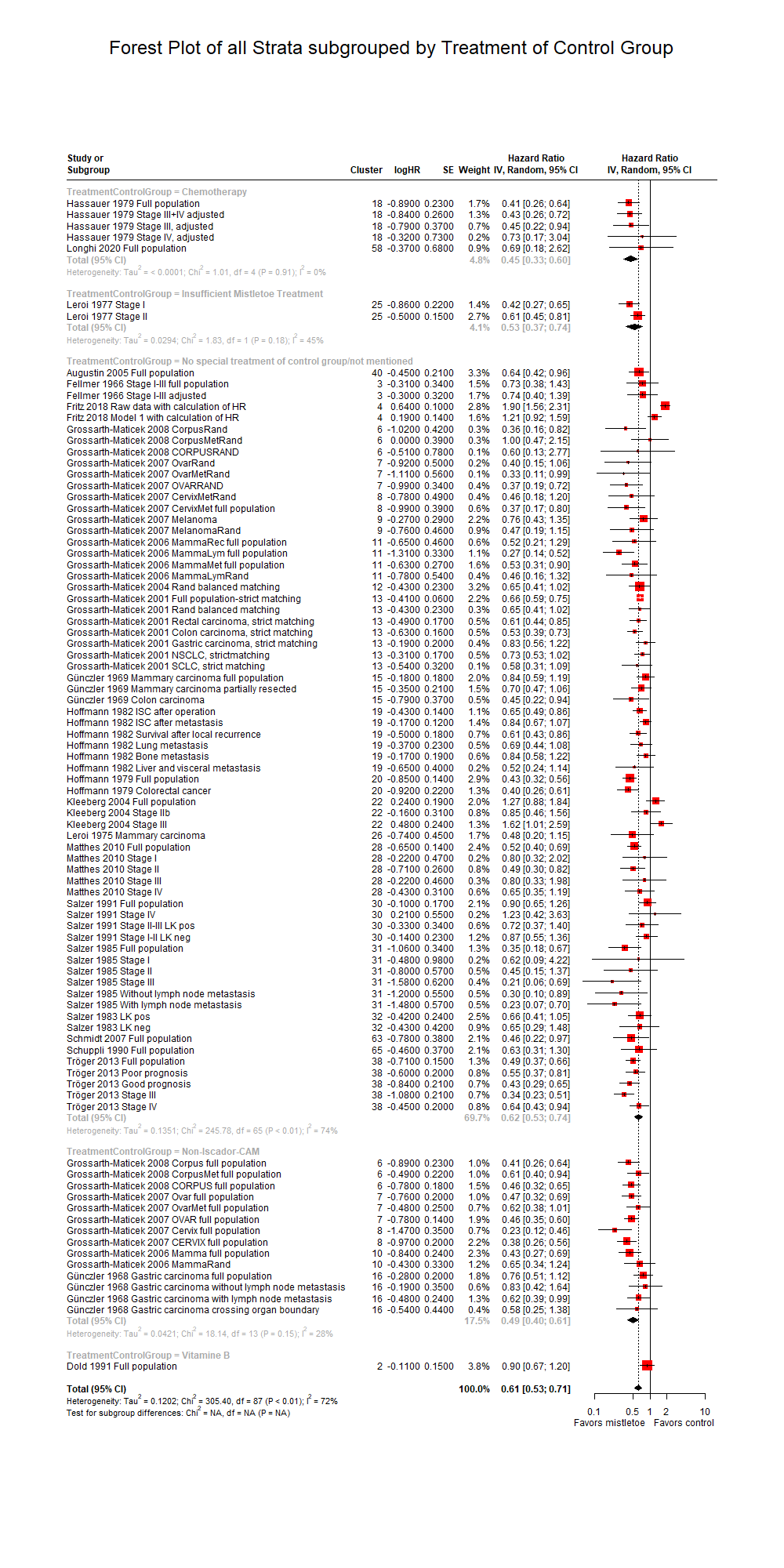

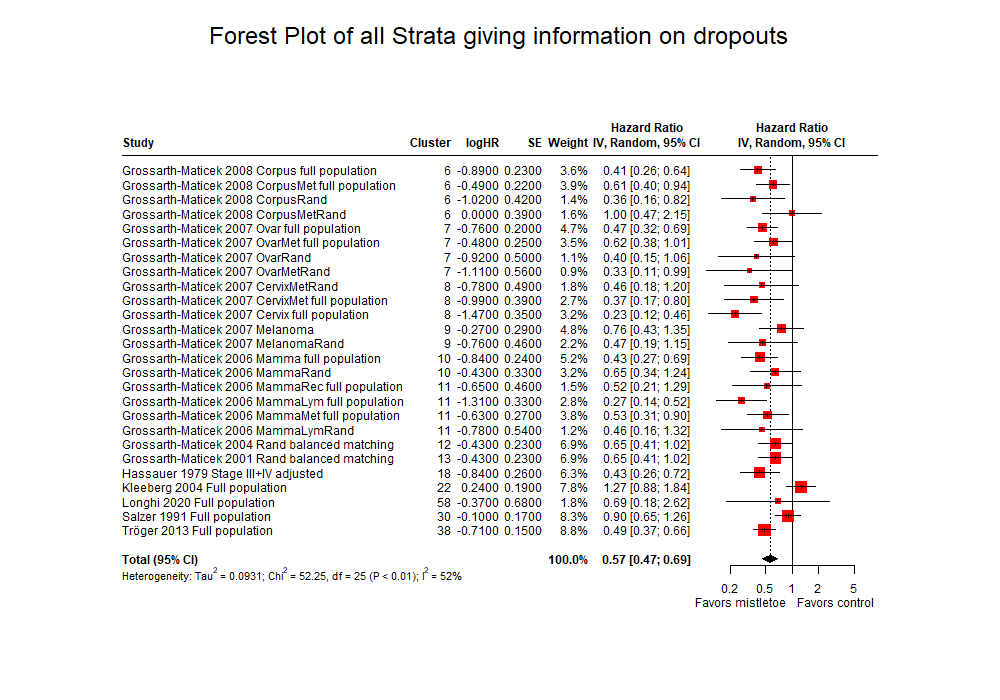

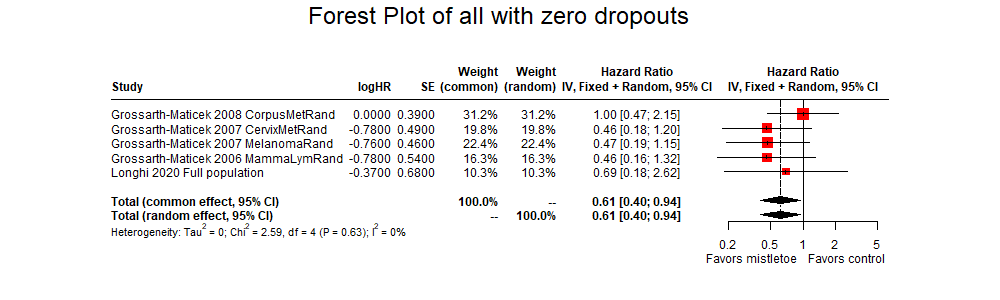

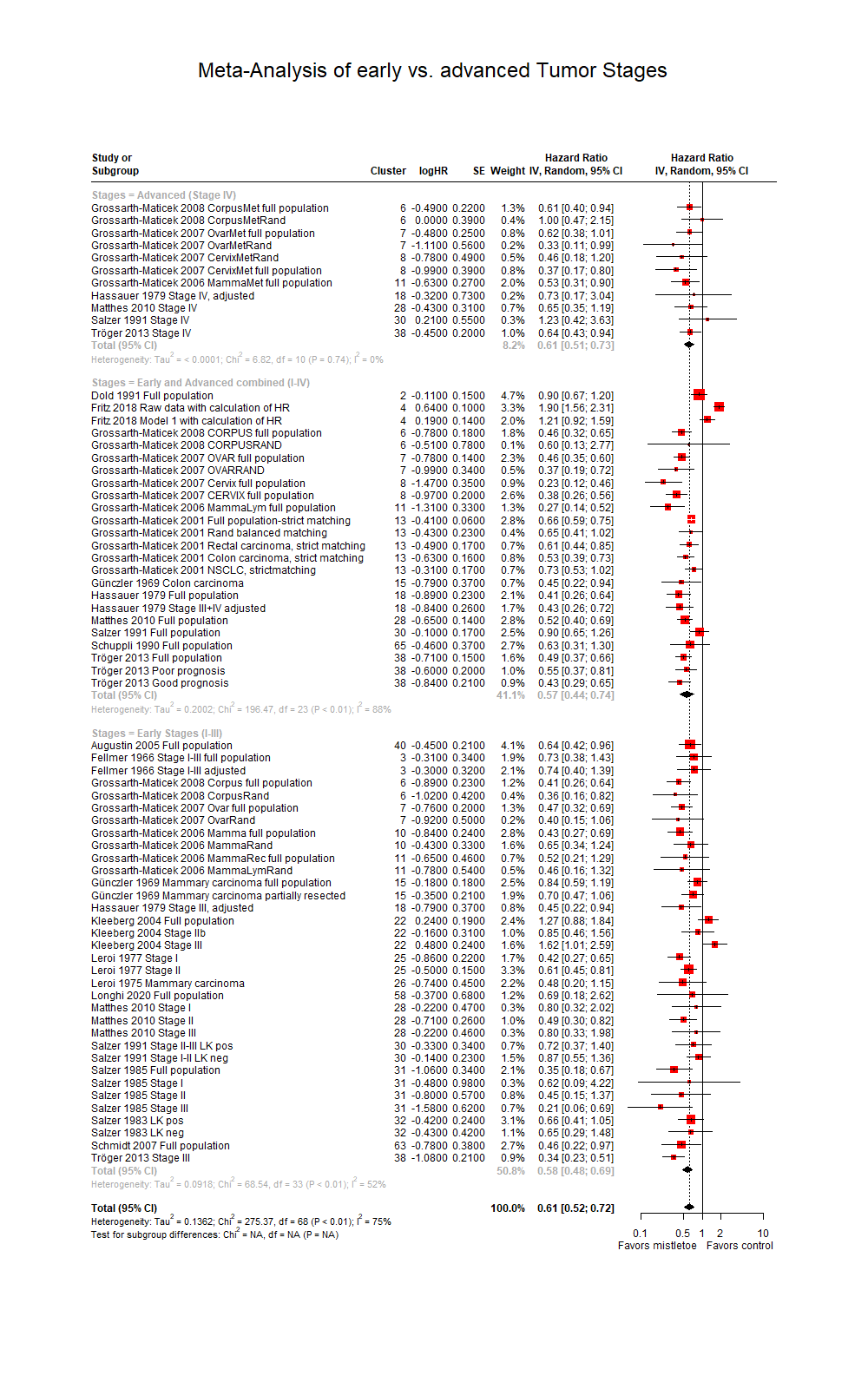

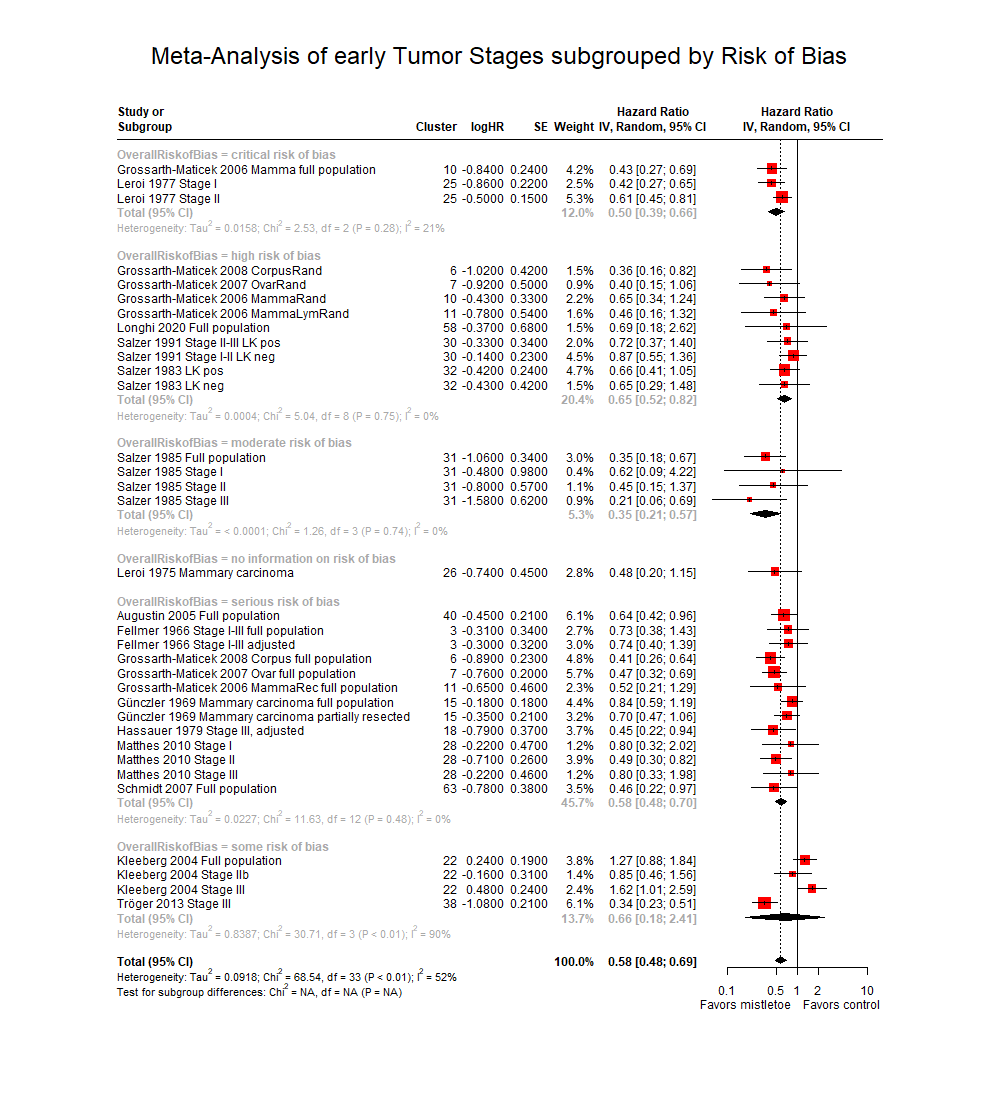

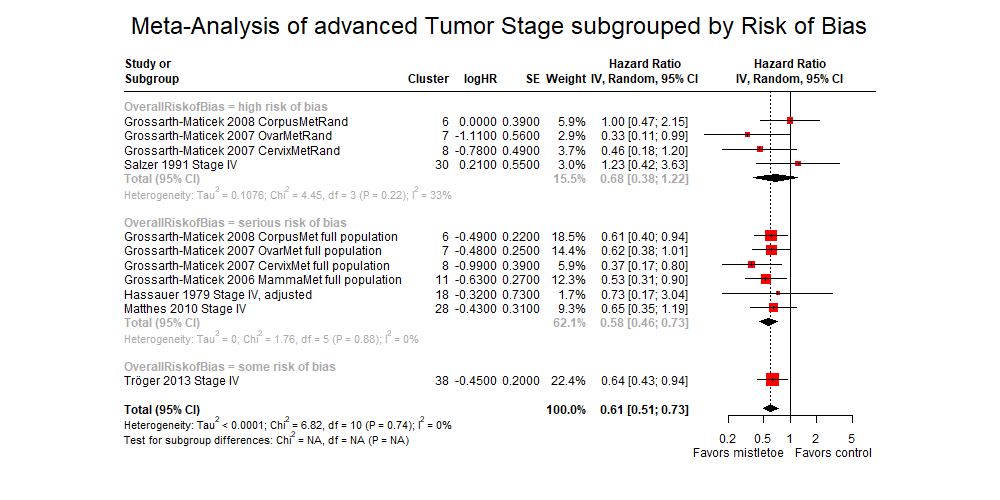

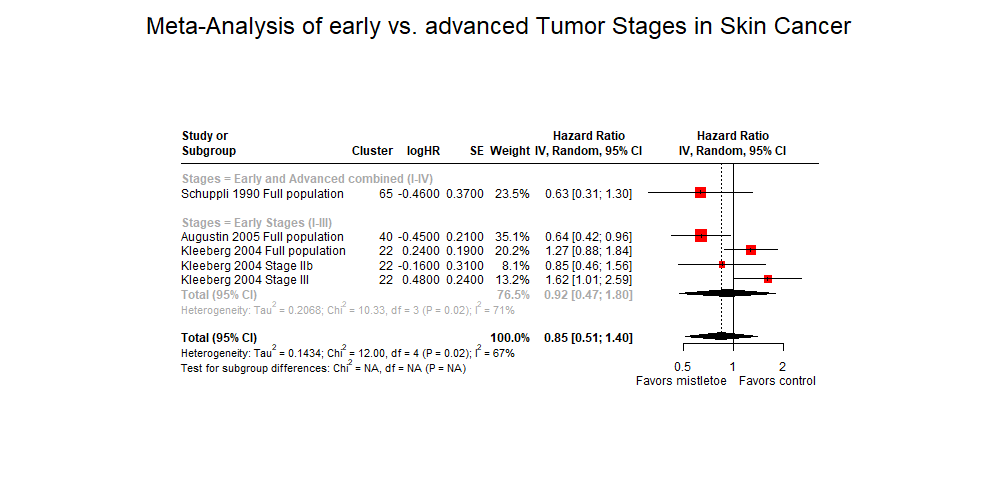

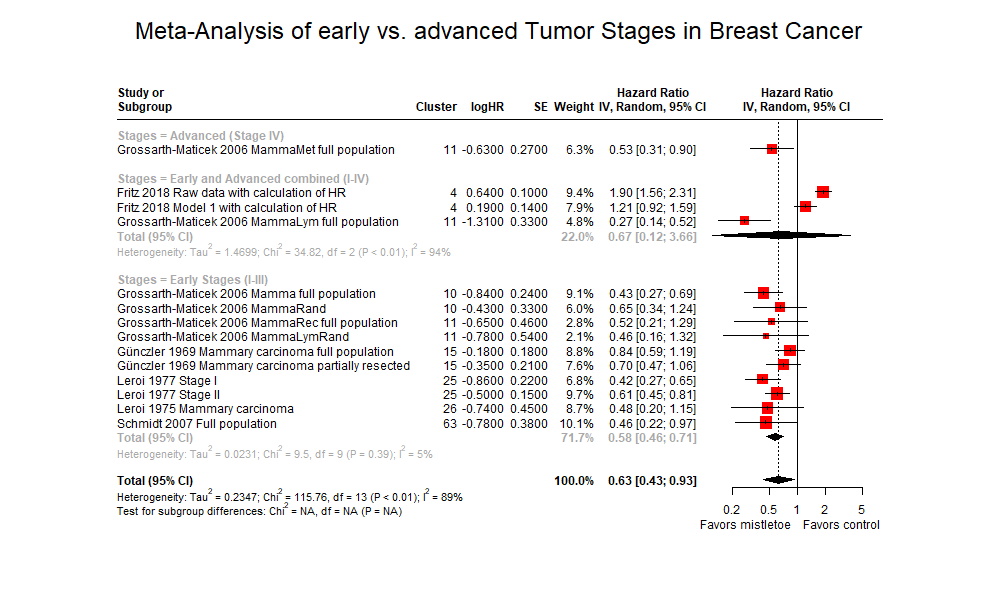

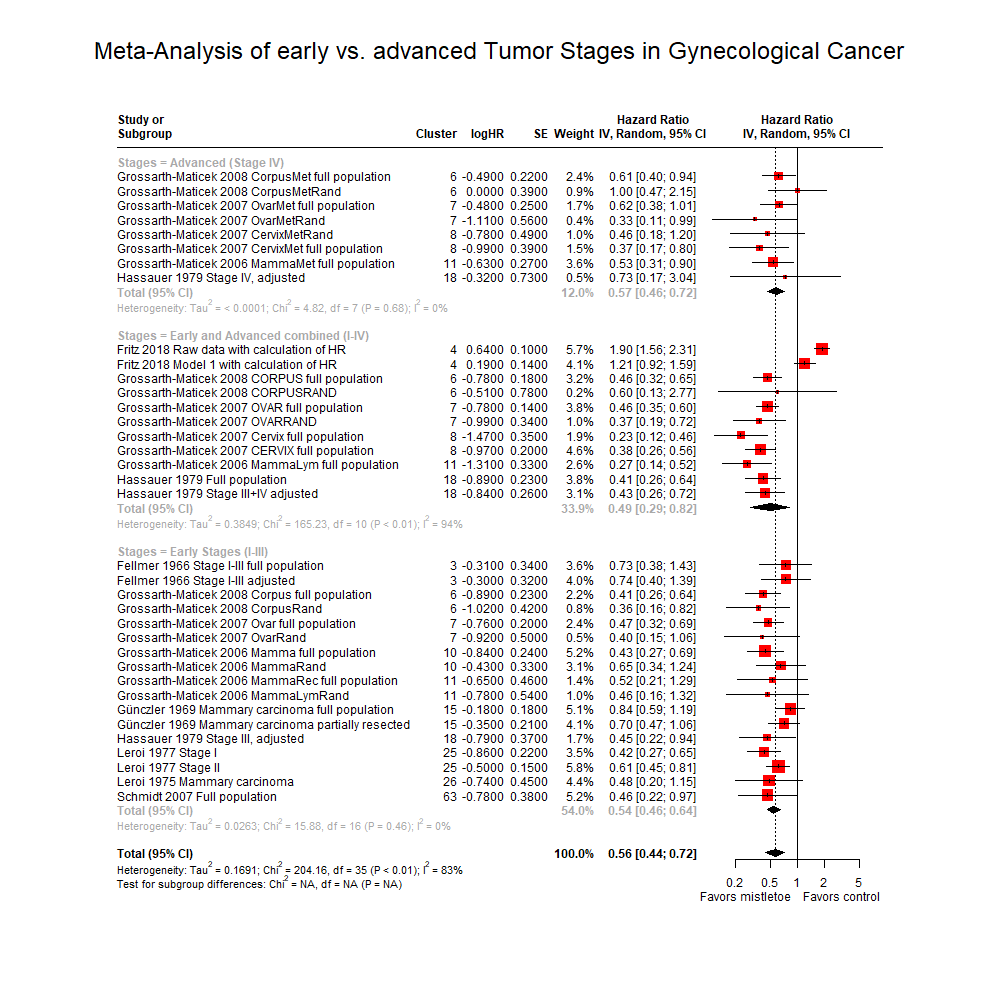

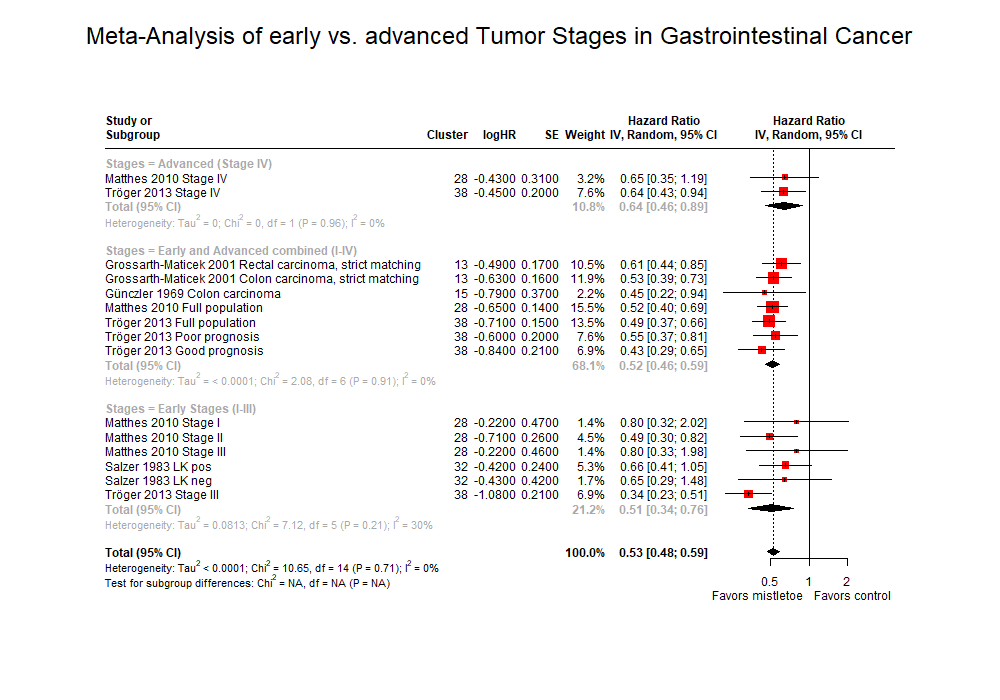

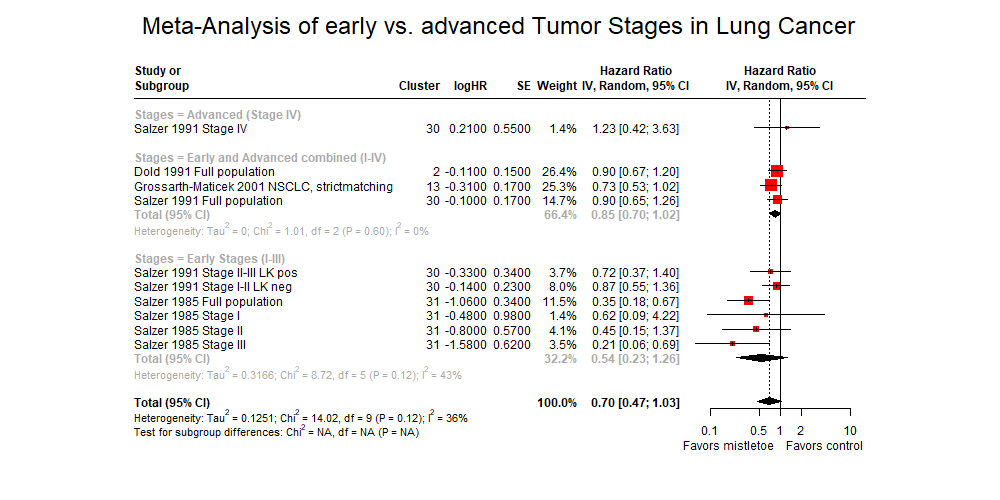

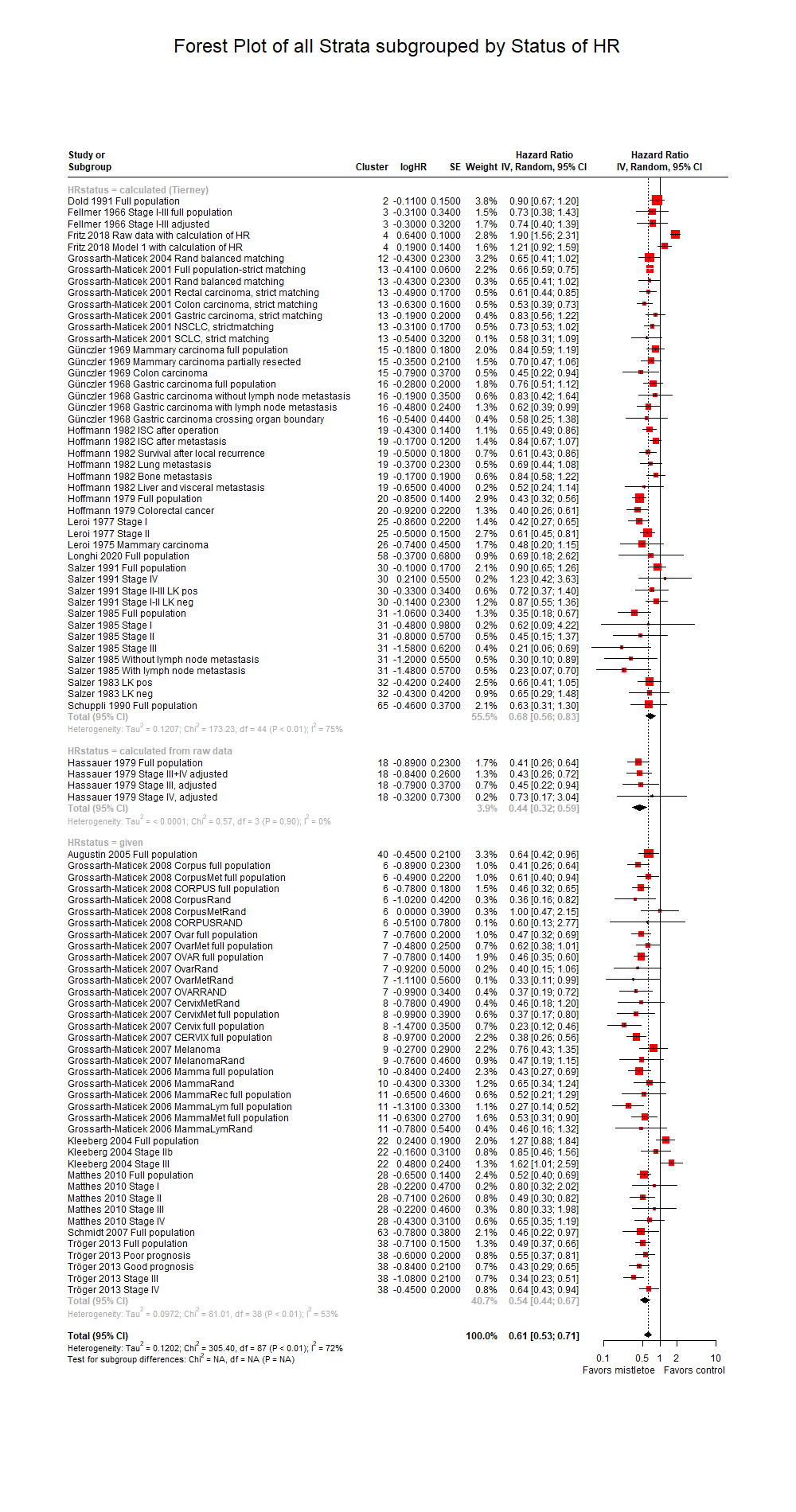


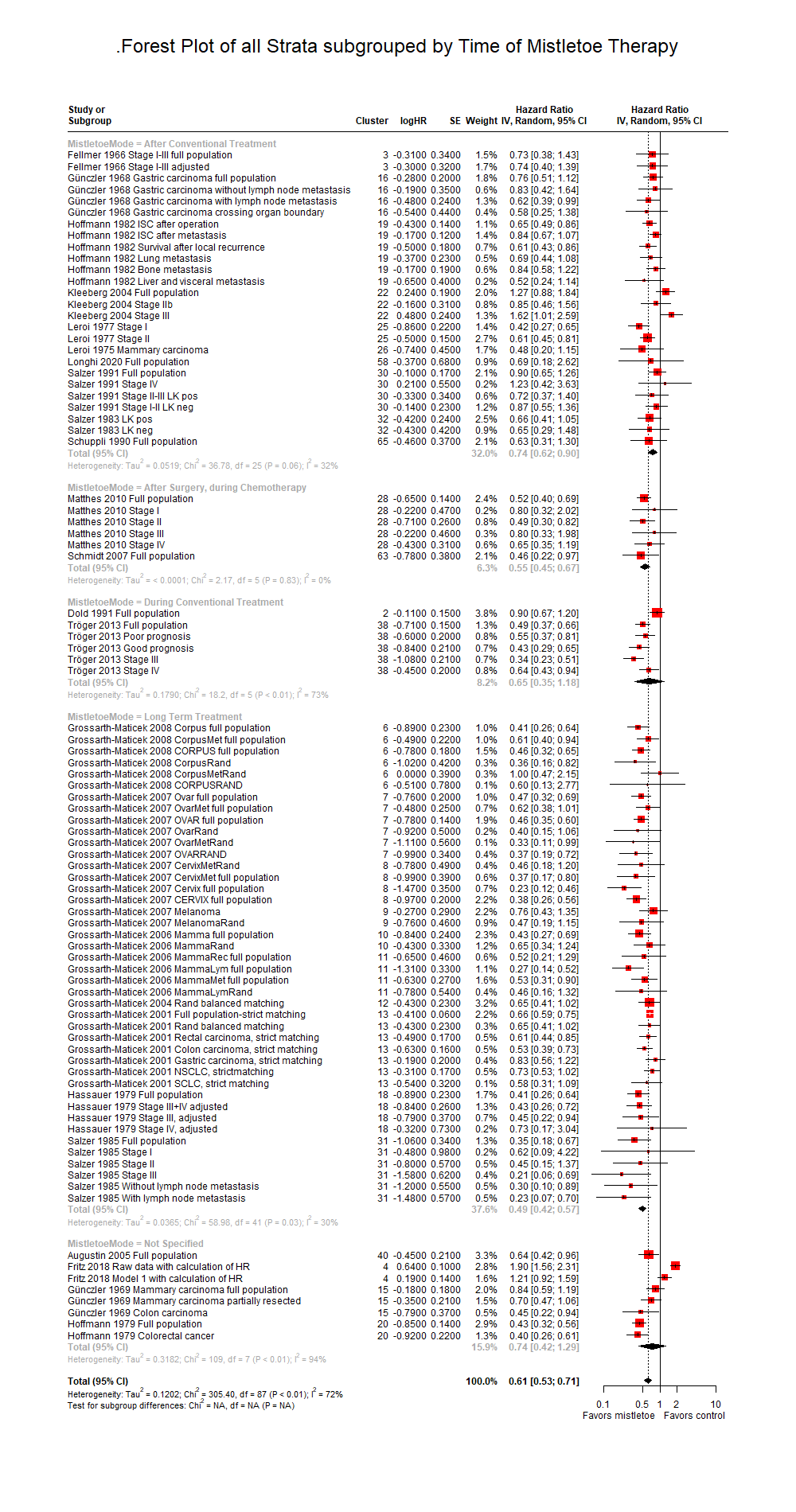

Supplement: Supplementary file 4 — Supplementary file4 (DOCX 1204 KB) [file 432_2024_5742_MOESM4_ESM.docx]
